# Genome-wide association and dissociation studies in *Pantoea ananatis* reveal potential virulence factors affecting *Allium porrum* and *Allium fistulosum*  ×  *Allium cepa* hybrid

**DOI:** 10.3389/fmicb.2022.1094155

**Published:** 2023-02-02

**Authors:** Brendon K. Myers, Gi Yoon Shin, Gaurav Agarwal, Shaun P. Stice, Ronald D. Gitaitis, Brian H. Kvitko, Bhabesh Dutta

**Affiliations:** ^1^Department of Plant Pathology, The University of Georgia, Tifton, GA, United States; ^2^Department of Plant Pathology, The University of Georgia, Athens, GA, United States

**Keywords:** *Pantoea*, *Allium*, genome-wide-association, gene-pair, virulence

## Abstract

*Pantoea ananatis* is a member of a *Pantoea* species complex that causes center rot of bulb onions (*A. cepa*) and also infects other *Allium* crops like leeks (*Allium porrum*), chives (*Allium schoenoprasum*), bunching onion or Welsh onion (*Allium fistulosum*), and garlic (*Allium sativum*). This pathogen relies on a chromosomal phosphonate biosynthetic gene cluster (HiVir) and a plasmid-borne thiosulfinate tolerance cluster (*alt*) for onion pathogenicity and virulence, respectively. However, pathogenicity and virulence factors associated with other *Allium* species remain unknown. We used phenotype-dependent genome-wide association (GWAS) and phenotype-independent gene-pair coincidence (GPC) analyses on a panel of diverse 92 *P. ananatis* strains, which were inoculated on *A. porrum* and *A. fistulosum* × *A. cepa* under greenhouse conditions. Phenotypic assays showed that, in general, these strains were more aggressive on *A. fistulosum* × *A. cepa* as opposed to *A. porrum*. Of the 92 strains, only six showed highly aggressive foliar lesions on *A. porrum* compared to *A. fistulosum* × *A. cepa*. Conversely, nine strains showed highly aggressive foliar lesions on *A. fistulosum* × *A. cepa* compared to *A. porrum*. These results indicate that there are underlying genetic components in *P. ananatis* that may drive pathogenicity in these two *Allium* spp. Based on GWAS for foliar pathogenicity, 835 genes were associated with *P. ananatis*’ pathogenicity on *A. fistulosum* × *A. cepa* whereas 243 genes were associated with bacterial pathogenicity on *A. porrum*. The Hivir as well as the *alt* gene clusters were identified among these genes. Besides the ‘HiVir’ and the *alt* gene clusters that are known to contribute to pathogenicity and virulence from previous studies, genes annotated with functions related to stress responses, a potential toxin-antitoxin system, flagellar-motility, quorum sensing, and a previously described phosphonoglycan biosynthesis (pgb) cluster were identified. The GPC analysis resulted in the identification of 165 individual genes sorted into 39 significant gene-pair association components and 255 genes sorted into 50 significant gene-pair dissociation components. Within the coincident gene clusters, several genes that occurred on the GWAS outputs were associated with each other but dissociated with genes that did not appear in their respective GWAS output. To focus on candidate genes that could explain the difference in virulence between hosts, a comparative genomics analysis was performed on five *P. ananatis* strains that were differentially pathogenic on *A. porrum* or *A. fistulosum* × *A. cepa*. Here, we found a putative type III secretion system, and several other genes that occurred on both GWAS outputs of both *Allium* hosts. Further, we also demonstrated utilizing mutational analysis that the *pepM* gene in the HiVir cluster is important than the *pepM* gene in the pgb cluster for *P. ananatis* pathogenicity in *A. fistulosum* × *A. cepa* and *A. porrum*. Overall, our results support that *P. ananatis* may utilize a common set of genes or gene clusters to induce symptoms on *A. fistulosum × A. cepa* foliar tissue as well as *A. cepa* but implicates additional genes for infection on *A. porrum*.

## Introduction

*Pantoea ananatis* is one of several species of bacteria within the *Pantoea* genus that causes onion center rot. Onion center rot can cause considerable losses in both yield and quality in *Alliums*, particularly in bulb onions (*Allium cepa*) in the southeastern United States (Gitaitis et al., 2003; Stice et al., 2018; Stumpf et al., 2018). There is currently no known resistance to *P. ananatis* in commercial onion cultivars and resistance in other *Allium* spp. is yet to be evaluated. *P. ananatis* invades the plant through foliar wounds leading to water-soaked lesions, blighting, and wilting of the leaf. Foliar colonization can lead to bulb invasion that often results in further post-harvest losses (Carr et al., 2013). While *P. ananatis* can be seedborne and seedling-transmitted, thrips (*Frankliniella fusca*, *Thrips tabaci*) mediated transmission seems to be more common and epidemiologically important, particularly in Georgia, United States (Dutta et al., 2014a, 2016). Several published reports indicate that these thrips species can acquire epiphytic *P. ananatis* populations from various environmental host plants including weeds and can transmit the pathogen to healthy onion seedlings (Dutta et al., 2014a, 2016). *P. ananatis* collectively has a broad host range as it can cause disease in a diverse range of crops including maize (*Zea mays L*.), pineapple (*Ananas comosus*), rice (*Oryza sativa*), and Sudan grass (*Sorghum bicolor × S. bicolor var. Sudanese*) (De Maayer et al., 2014).

Based on observations made by Stice et al. (2018), *P. ananatis* is pathogenic on a variety of *Allium* spp.; however, the authors observed that the strains varied greatly in their pathogenic potential and aggressiveness on onion, shallot (*A. cepa* var. *aggregatum*), chives (*A. schoenoprasum*), and leeks (*A. porrum*). The bunching onion or Welsh onion (*A. fistulosum*) has been shown to be a host for *P. ananatis* (Kido et al., 2010; Wang et al., 2018). Symptoms observed on these hosts were comparable to those observed on typical bulb-forming onion seedlings. *P. ananatis* is unusual when compared with other Gram-negative plant pathogenic bacteria in that it does not utilize either the type II secretion system (T2SS) or the type III secretion system (T3SS) to secrete cell-wall degrading enzymes and deliver virulence effectors into the target host cell, respectively (Chang et al., 2014). Asselin et al. (2018) and Takikawa and Kubota (2018) independently identified a chromosomally located “HiVir,” or High Virulence gene cluster. This gene cluster has been demonstrated to be a critical pathogenicity factor for *P. ananatis* in onions (Asselin et al., 2018; Takikawa and Kubota, 2018). The HiVir gene cluster encodes for a phosphonate phytotoxin “pantaphos” that was shown to be critical for bulb necrosis (Asselin et al., 2018; Polidore et al., 2021). Another cluster of importance was recently characterized by Stice et al. and is a plasmid-borne virulence gene cluster coined as “*alt*” or thiosulfinate (**Al**licin) **t**olerance (Stice et al., 2018, 2020, 2021). The *alt* cluster in *P. ananatis* imparts tolerance to thiosulfinates allowing *P. ananatis* to colonize the thiosulfinate-rich environment in necrotic onion bulbs (Stice et al., 2018, 2020, 2021. Despite these advances in understanding pathogenicity and virulence mechanisms in the *P. ananatis*-onion pathosystem, the mechanisms behind bacterial capacity to colonize other *Allium* spp. such as *A. porrum* and *A. fistulosum × A. cepa* remain unknown.

Whole-genome sequencing (WGS) of bacteria is routinely performed in many laboratories for diagnostics, understanding host-pathogen interactions, and for ecological studies (Farhat et al., 2013; Sheppard et al., 2013; Chewapreecha et al., 2014; Hall, 2014; Laabei et al., 2014; Holt et al., 2015; Desjardins et al., 2016; Earle et al., 2016; Lees et al., 2016). Despite generation of large informatics data sets, the primary challenge when handling these methodologies is managing an appropriate strategy to go from raw data to detailed, biologically relevant information. One strategy commonly used to relate genotype to phenotype is the genome-wide association study (GWAS). This strategy was first adopted in human-based medicine, but soon after gained general popularity in analyzing bacterial genomes to answer various questions related to pathogenicity, antibiotic resistance, and bacterial survival (Farhat et al., 2013; Sheppard et al., 2013; Chewapreecha et al., 2014; Hall, 2014; Laabei et al., 2014; Holt et al., 2015; Desjardins et al., 2016; Earle et al., 2016; Lees et al., 2016). Genome-wide association has proven to be an excellent tool in correlating genomic variations with observed phenotypes such as virulence factors, antibiotic resistance, and tolerance to abiotic and biotic stresses (Farhat et al., 2013; Sheppard et al., 2013; Chewapreecha et al., 2014; Hall, 2014; Laabei et al., 2014; Holt et al., 2015; Desjardins et al., 2016; Earle et al., 2016; Lees et al., 2016). This trend is no different for topics in plant pathology either. For example, GWAS can be utilized for the determining resistance to pathogens through quantitative trait locus mapping (Thoen et al., 2016) or to identify candidate pathogenic genetic determinants in plant pathogens (Dalman et al., 2013; Monteil et al., 2016; Bartoli and Roux, 2017). In this study, we utilized the WGS strategy to build a pan-genome and compared the combined genomes of bacterial strains to pathogenicity phenotype on two *Allium* spp. (*A. porrum* and *A. fistulosum × A. cepa*).

The complete complement of the total genes within a genomic set is termed as “pan-genome” (Medini et al., 2005; Tettelin et al., 2005). A pangenome consist of “core” genes that are common across all bacterial strains of a species and the “accessory” genes that are specific/present in only some strains (Tettelin et al., 2005). Accessory genes are responsible for key differentiation among strains and have been associated with pathogenicity islands or with niche adaptation (Brockhurst et al., 2019). Ideally, pan-GWAS can also be used to identify associations between genotypic traits and observed phenotype. This may aid in determining potential gene or gene clusters that are responsible for the observed phenotype with a pre-defined set of statistical criteria (Brynildsrud et al., 2016). In the current study, we utilized a pangenome-wide association study (pan-GWAS) to identify presence and absence variants in *P. ananatis* strains (*n* = 92) that are associated with foliar symptoms in *A. porrum* and *A. fistulosum × A. cepa*. Using pangenome-GWAS, we report a set of potential *P. ananatis* virulence factors associated with these Allium hosts including the “HiVir” and the phosphonoglycan biosynthesis (pgb) clusters, a type III secretion system, a putative toxin/antitoxin region, and several other virulence-associated genes. These include the phosphonoglycan biosynthesis (pgb) cluster, a type III secretion system, a putative toxin/antitoxin region, and several other virulence-associated genes. Further, we also demonstrated that the *pepM* gene in the HiVir cluster is important than the *pepM* gene in the “pgb” cluster for *P. ananatis* pathogenicity in *A. fistulosum × A. cepa* and *A. porrum*.

A recent study tested the hypothesis that genes generally co-occur (associate) or avoid each other (dissociate) based on the fitness consequences in a particular set of genomes (Whelan et al., 2019). For example, in our case, we presume that genes, which allow necrosis in *Allium* spp. and confer thiosulfate tolerance should associate as these traits are co-beneficial for survival in the niche of an *Allium* host. However, genes that, in combination, result in the production of a toxic byproduct (as has been observed with siderophore biosynthesis in *Salinispora* spp.; Bruns et al., 2018) or perform some redundant function, or trigger an immune response, should dissociate with each other as co-expression may reduce bacterial fitness. Therefore, we analyzed genomic interactions of accessory genes in the pangenome derived from 92 *P. ananatis* genomes to determine genes associated with virulence, with a premise that virulence genes should associate with other virulence genes throughout the accessory pangenome and that redundant virulence genes should naturally dissociate.

## Materials and methods

### Bacterial strains, identification, culturing, and mutagenesis

*Pantoea ananatis* strains (*n* = 92) used in this study were isolated from diverse sources; weeds, thrips, and onion tissue (foliage, bulbs, and seeds) in Georgia from 1992 to 2019. The metadata for each strain such as the source, year of isolation, and county of origin in Georgia for these strains and their distribution within each category are listed (Table 1).

**Table 1:** *Pantoea ananatis* strains, their source of isolation, and their associated pathogenicity and aggressiveness on leek (*Allium porrum*) and Japanese bunching onion (*Allium fistulosum × Allium cepa*) and their phenotype on red onion scale.

Strain	Source		Leek (*Allium porrum* cv. King Richard)^a^	Bunching onion (*A. fistulosum × A. cepa*; cv. Guardsman)^b^	Red onion scale assay^c^
PNA_15_3^d^	Onion	Tattnall Co. GA	++^*^	–	–
PANS_99_14^d^	*Digitaria* spp.	Tift Co. GA	+	–	–
PANS_99_11^e^	*Digitaria* spp.	Tift Co. GA	+++	++	+
PANS_99_12^e^	*Digitaria* spp.	Tift Co. GA	+++^*^	+	+
PNA_06_4^e^	Onion	Wayne Co. GA	+++	++	+
PNA_97_1^e^	Onion	Tift Co. GA	+++^*^	++	+
PNA_99_9^e^	Onion seedlings	Tattnall Co. GA	++	+++	+
PNA_99_7^e^	Onion leaf	Tattnall Co. GA	+	+++	+
PNA_99_2^e^	Onion leaf	Tattnall Co. GA	+	+++	+
PNA_99_14^e^	Onion seedlings	Toombs Co. GA	+^*^	+++	+
PNA_98_1^e^	Onion	Tattnall Co. GA	+	+++	+
PNA_97_11^e^	Onion	Toombs Co. GA	+	+++^*^	+
PANS_02_7^e^	Thrips from peanut blossoms	Tift Co. GA	+	+++	+
PANS_02_6^e^	Thrips from peanut blossoms	Tift Co. GA	+^*^	+++	+
PNA_99_3^e^	Onion seedlings	Tift Co. GA	+	+++	+
PNA_07_10^f^	Onion	Toombs Co. GA	–	+	+
PNA_07_1^f^	Onion	Tattnall Co. GA	–	+	+
PNA_05_1^f^	Onion	Vidalia Region, GA	–	+	+
PNA_03_2^f^	Onion	Tift Co. GA	–	+^*^	–
PNA_03_1^f^	Onion	Tift Co. GA	–	+	+
PNA_02_12^f^	Onion	Tift Co. GA	–	+^*^	+
PANS_99_36^f^	*Richardia scabra* L.	Terrell Co. GA	–	+^*^	–
PANS_99_31^f^	*Urochloa texana*	Tattnal Co. GA	–^*^	+^*^	+
PANS_99_29^f^	*Digitaria* spp.	Tift Co. GA	–^*^	+	+
PANS_99_27^f^	*Desmodium tortuosum*	Vidalia Region, GA	–	+	+
PANS_99_25^f^	*Acanthospermum hispidum*	Vidalia Region, GA	–	+^*^	+
PANS_200_1^f^	*Slender amaranth*	Reidsville, GA	–	+^*^	–
PNA_18_8S^f^	Onion	Vidalia Region, GA	–	+	–
PNA_18_7S^f^	Onion	Vidalia Region, GA	–	+	+
PNA_97_3^f^	Onion	Toombs Co. GA	–	+	+
PNA_98_7^f^	Onion	Tift Co. GA	–	+	–
PNA_98_3^f^	Onion	Dougherty, GA	–^*^	+	–
PNA_11_1^f^	Onion	Vidalia Region, GA	–	+^*^	–
PNA_08_1^f^	Onion	Tattnall Co. GA	–^*^	++	+
PNA_07_14^f^	Onion	Toombs Co. GA	–	+^*^	–
PANS_02_1^f^	Adult tobacco thrips from peanut	Tift Co. GA	–	+^*^	–
PANS_01_2^f^	Thrips from Onion leaf	Tift Co. GA	–	+^*^	+
PANS_19_2^f^	*Digitaria* spp.	Tift Co. GA	–	+	+
PANS_19_6^f^	*Richardia scabra* L.	Tift Co. GA	–	+	+
PANS_19_17^f^	*Richardia scabra* L.	Tift Co. GA	–	+^*^	–
PNA_18_2^g^	Onion	Vidalia Region, GA	++	+	+
PNA_15_1^g^	Onion	Tattnall Co. GA	++	+	+
PANS_200_2^g^	*Portulaca* spp.	Reidsville, GA	++^*^	+^*^	+
PANS_01_6^g^	Adult tobacco thrips	Tift Co. GA	++	+	+
PANS_01_5^g^	Adult tobacco thrips	Tift Co. GA	++^*^	+^*^	+
PANS_19_12^g^	*Verbena bonariensis*	Tift Co. GA	++^*^	+	–
PANS_19_13^g^	*Verbena bonariensis*	Tift Co. GA	++	+^*^	–
PANS_02_5^g^	Thrips from peanut blossoms	Tift Co. GA	++	++	+
PNA_97_2	NA	NA	+^*^	+	–
PNA_99_8	Onion leaf	Wheeler Co. GA	+^*^	++	+
PNA_99_6	Onion leaf	Toombs Co. GA	+	++	+
PNA_99_1	Onion	MT Vernon, GA	+	++	+
PNA_98_8	Onion	Vidalia Region GA	+	++	+
PNA_98_2	Onion	Tift Co. GA	+^*^	++	+
PNA_98_12	Onion	Toombs Co. GA	+	++	+
PNA_98_11	Onion	Evans Co. GA	+^*^	++^*^	–
PNA_92_7	Onion	Vidalia Region GA	+^*^	+	+
PNA_200_7	Onion	Tift Co. GA	+^*^	+	+
PNA_200_12	Onion	Tift Co. GA	+^*^	+	+
PNA_200_11	Onion	Tift Co. GA	+	++^*^	+
PNA_200_10	Onion	Tift Co. GA	+^*^	+	+
PNA_18_9S	Onion	Vidalia Region, GA	+^*^	+^*^	+
PNA_18_5S	Onion	Vidalia Region, GA	+	+^*^	+
PNA_18_5	Onion	Vidalia Region, GA	+^*^	+	+
PNA_18_3S	Onion	Vidalia Region, GA	+^*^	+^*^	+
PNA_18_1	Onion	Vidalia Region, GA	+	+	+
PNA_07_7	Onion	Toombs Co. GA	+^*^	+	+
PNA_07_13	Onion	Toombs Co. GA	+^*^	+	–
PANS_99_33	*Richardia scabra* L.	Coffee Co. GA	+	++^*^	+
PANS_99_26	*Euphorbia hyssopifolia*	Vidalia Region, GA	+	+	–
PANS_99_22	*Digitaria* spp.	Tift Co. GA	+	+	–
PANS_02_8	Thrips from peanut leaf	Tift Co. GA	+^*^	++	+
PANS_99_18	*Richardia scabra* L.	Tift Co. GA	+^*^	+	+
PANS_02_12	Peanut leaf	Tift Co. GA	+	+	–
PANS_19_11	*Richardia scabra* L.	Tift Co. GA	+	+	+
PNA_06_1	Onion	Vidalia Region, GA	+	+	–
PANS_04_1	Thrips	Tift Co. GA	–	–^*^	–
PANS_99_24	Onion seedlings	Vidalia region	–	–^*^	–
PANS_19_8^h^	*Richardia scabra* L.	Tift Co. GA	–	–	+
PANS_19_10^h^	*Richardia scabra* L.	Tift Co. GA	–	–	+
PNA_200_8^h^	Onion	Tift Co. GA	–^*^	–	–
PNA_200_3^h^	Onion	Tift Co. GA	–	–	–
PNA_18_6S^h^	Onion	Vidalia Region, GA	–	–	–
PNA_18_10S^h^	Onion	Vidalia Region, GA	–^*^	–	–
PNA_18_10^h^	Onion	Vidalia Region, GA	–^*^	–	–
PNA_14_2^h^	Onion	Lyons, GA	–	–^*^	–
PNA_13_1^h^	Onion	Lyons, GA	–	–^*^	–
PANS_99_23^h^	*Cyperus esculentus*	Vidalia Region, GA	–	–	–
PANS_04_2^h^	Adult tobacco thrips from peanut	Tift Co. GA	–	–	–
PANS_01_8^h^	Adult tobacco thrips	Tift Co. GA	–	–^*^	–
PANS_01_10^h^	Thrips feces from peanut leaf	Tift Co. GA	–	–^*^	–
PANS_99_10^h^	*Verbena bonariensis*	Tift Co. GA	–	–^*^	–
PANS_19_20^h^	*Verbena bonariensis*	Tift Co. GA	–	–	–

a Foliar lesion rating of *P. ananatis* strains on Leek (*A. porrum* cv. King Richard). Strains with a lesion length 0.2–0.5 cm, 0.5–0.96 cm and > 1 cm were considered as less aggressive (+), moderately aggressive (++), and highly aggressive (+++), respectively.

b Foliar lesion rating of *P. ananatis* strains on bunching onion (*A. fistulosum × A. cepa* cv. Guardsman). Strains with lesion lengths of < 0.7 cm, 0.7–1.4 cm and > 1.4 cm were regarded as less aggressive (^+^), moderately aggressive (^++^), and highly aggressive (^+++^), respectively.

c Ability of strain to clear red anthocyanin pigment and cause pitting on onion scales.

d Strains that are highly aggressive on leeks but non-pathogenic on bunching onion.

e Strains that are highly aggressive on leeks and bunching onions and are able to cause necrosis on red-onion scale.

f Strains that are non-pathogenic on leeks and less-aggressive on bunching onion.

g Strains that are moderately aggressive on leeks, and are less-aggressive on bunching onions.

h Non-pathogenic strains.

* Lesion phenotype was inconsistent among the six replicates.

**Figure 1:**
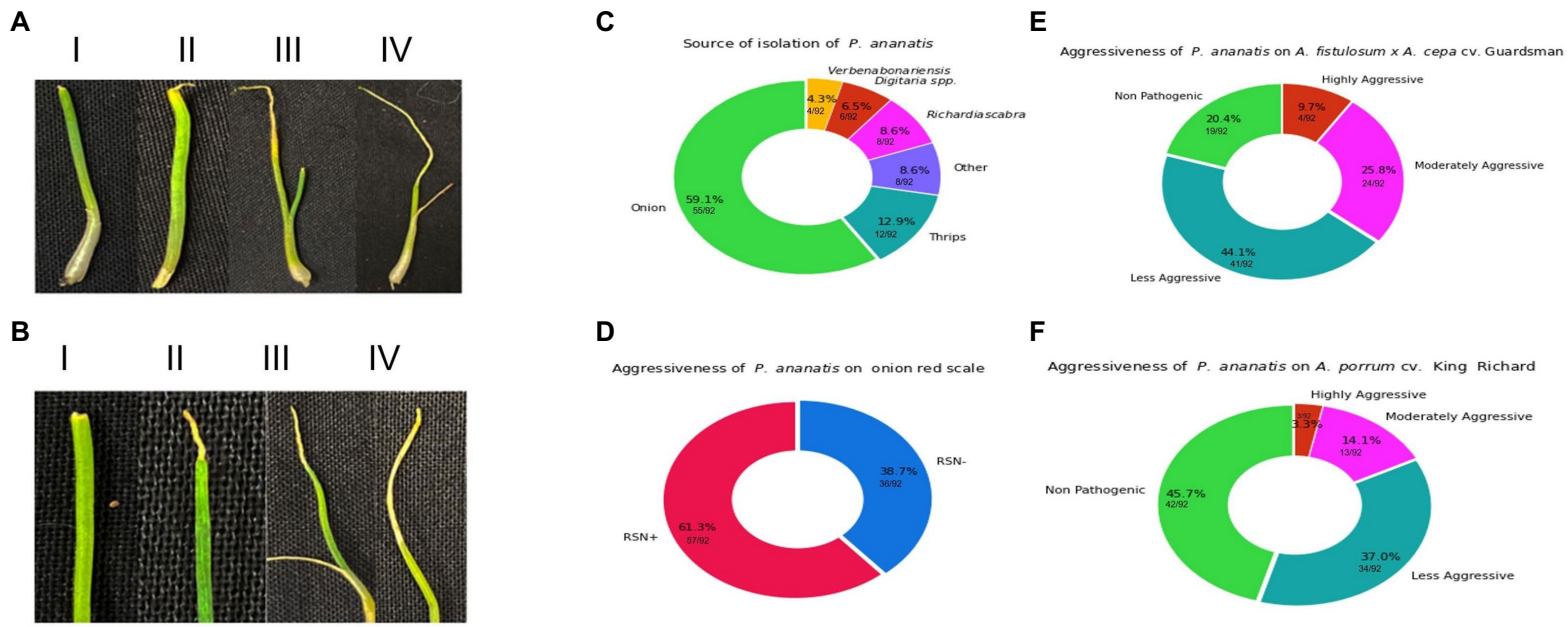
Visual representation of spectrum of foliar symptoms caused by *Pantoea ananatis* inoculation on *Allium porrum* (cv. King Richard) and *Allium fistulosum* × *Allium cepa* (cv. Guardsman) as well as source of isolation and results the red-onion scale necrosis (RSN) assay. **(A)** Shows examples of foliar lesion severity and strain aggressiveness on *A. fistulosum* × *A. cepa*. The panels in **A** (i–iv) indicate symptoms associated with increasing level of aggressiveness. **(B)** Shows examples of foliar lesion severity and strain aggressiveness on *A. porrum*. The panels in **B** (i–iv) indicate symptoms associated with increasing level of aggressiveness. **(C)** shows the distribution of the sources of isolation of strains, where *A. cepa* (onions) contributes majority of the strains (59.1%) and strains from non-onion sources comprise of 40.9% (*Richardia scabra*: 8.6%, *Digitaria* spp.: 6.5%, *Verbena bonariensis*: 4.3%, thrips: 12.9%, and other sources: 8.6%). **(D)** Displays the percentage of strains that can cause red-onion scale necrosis (61.3%) and those that could not (38.7%). **(E)** (*A. fistulosum* × *A. cepa*) and **(F)** (*A. porrum*) visualize the breakdown of strains that are either highly- or moderately- or less-aggressive or non-pathogenic on both *Allium* hosts.

Among the strains used, 55 strains (59.8%) were isolated from onion foliage or bulb tissue, which constituted the majority of the strains. The remaining 38 strains (40.2%) were isolated from other diverse sources including weeds, and thrips. This is followed by the weeds *Richardia scabra* L. (8.7%; 8/92), *Digitaria* spp. (6.5%; 6/92), and *Verbena bonariensis* (4.3%; 4/92). The strains from various plant sources constituted 8.7% (8/92) of the total strains studied (Table 1; Figure 1B). Strains from thrips (*Frankliniella fusca* and *F. occidentalis*) constituted the remaining 13% (12/92). These curated strains were initially identified as *P. ananatis* by their colony morphology and physiological characteristics such as being: Gram-negative, facultatively anaerobic, positive for indole production, and negative for nitrate reductase and phenylalanine deaminase. Further confirmation was done using a *P. ananatis*-specific PCR assay as described earlier (Walcott et al., 2002).

*Pantoea ananatis* inoculum for foliar pathogenicity phenotyping was prepared by transferring single colonies of each bacterial strain from 24 h-old cultures on nutrient agar (NA) medium to nutrient broth (NB). The broth was shaken overnight on a rotary shaker (Thermo Scientific, Gainesville, FL, United States) at 180 rpm. After 12 h of incubation, 1 ml of each bacterial suspension were centrifuged at 5000 × *g* (Eppendorf, Westbury, NY, United States) for 2 min. The supernatant was discarded, and the pellet was re-suspended in deionized water. Inoculum concentration was adjusted using a spectrophotometer (Eppendorf, Westbury, NY, United States) to an optical density of 0.3 at 600 nm [≈ 1 × 10^8^ colony forming unit (CFU)/ml].

Deletion of the *P. ananatis* PANS 02-18 *pepM_pgb_ pepM_HiVir_* genes was conducted by two-step allelic exchange as described by Stice et al. (2020). In brief, approximately 400-bp flanking regions to the targeted genes were directly synthesized as a single joined sequence by Twist bioscience and cloned *via* BP clonase II using primer-introduced *att*B1/2 recombination sites into the pR6KT2G Gateway^®^ compatible sacB-based allelic exchange vector. These deletion constructs were introduced into PANS 02–18 *via* biparental conjugation with the RHO5 *E. coli* strain and single crossover events were recovered *via* gentamicin selection. Second crossover events were recovered *via* liquid sucrose counter-selection and identified by screening for backbone eviction based on loss of gentamicin resistance and the formation of white colonies on X-gluc. Deletion mutants were identified and confirmed based on PCR and amplicon sequencing using independent primers designed to amplify from genomic regions adjacent to the 400-bp deletion flanking regions.

### Phenotypic assessment of *Pantoea ananatis*: Red onion scale necrosis, foliar pathogenicity, and aggressiveness assay on *Allium porrum* and *Allium fistulosum × Allium cepa*

Pathogenic potential of *P. ananatis* strains were initially phenotyped on onion scale using a previously described red scale necrosis (RSN) assay (Stice et al., 2018). Red onions (cv. Red Burgundy) were surface sterilized with 70% ethanol and the outermost scale sliced to approximately 3 cm × 4 cm. The resulting scales were set on a sterile petri dish or on sterilized microtube trays, with the bottom covered with sterilized paper towels pre-moistened with distilled water. Each onion scale was then wounded *via* direct penetration with a sterilized needle and inoculated with 10 μl of approximately 1 × 10^6^ CFU/ml inoculum of *P. ananatis*. A known onion-pathogenic strain (PNA 97-1) was used as a positive control (Gitaitis and Gay, 1997). Sterile water was used as a negative control. The resulting petri dishes were then laid in an aluminum tray (46 cm × 25 cm × 10 cm) and covered with a plastic lid. These onion scales were then incubated for 5 days in the dark. The area of pigment clearing, and necrotic lesions were measured at 7 days post-inoculation. Strains that did not clear the red anthocyanin pigment or developed necrotic lesions, were declared non-pathogenic. Strains that caused necrosis along with pitting, with a visible zone of pigment clearing were considered pathogenic. Three replications were performed for each strain and in total two experiments were conducted.

Foliar pathogenicity and aggressiveness of *P. ananatis* strains (*n* = 92) were determined on *A. porrum* (cv. King Richard) and *A. fistulosum × A. cepa* (cv. Guardsman) under controlled greenhouse conditions. *P. ananatis* strain (PNA 97-1) was used as a positive control for both *Allium* species (14,31). Seedlings were established in plastic pots (T.O. plastics, Clearwater, MN) with dimension of 9 cm × 9 cm × 9 cm (length × breadth × height) containing a commercial potting mix (Sta-green, Rome, GA, United States). The seedlings were maintained under greenhouse condition at 25–28°C and 70–90% relative humidity with a light:dark cycle of 12:12 h. Osmocote smart release plant food (The Scotts Company, Marysville, OH, United States) was used for periodic fertilization. Bacterial strains were maintained on NA plates and inoculum was generated as described above. Once the primary leaf of each *Allium* spp. reached 9 cm, seedlings were inoculated using a cut-tip method as described previously (Dutta et al., 2014a). Briefly, a wound was created by cutting the central leaf (2 cm from the apex) with a sterile pair of scissors. Using a micropipette, a 10 μl drop of a bacterial suspension containing 1 × 10^8^ CFU/ml (1 × 10^6^ CFU/leaf) was deposited at the cut-end. Seedlings inoculated with sterile water as described above were used as negative control. Three replications per strain per host were used for one experiment and a total of two independent experiments was conducted. The seedlings were observed daily for symptom development until 5 days post-inoculation (DPI) and were compared with the foliar symptoms displayed by the positive control on each *Allium* species. The aggressiveness of *P. ananatis* strains was determined based on the lesion length on each *Allium* spp. For *A. porrum*, strains that caused a lesion length of 0.2–0.5 cm were considered less aggressive, 0.5–0.9 cm moderately aggressive, and > 1 cm highly aggressive. For *A. fistulosum × A. cepa*, strains were considered highly aggressive when a lesion length of > 1.4 cm was observed. Lesion lengths ranging from 0.7 to 1.4 cm were considered as moderately aggressive and strain with lesion length < 0.7 cm was regarded as less aggressive. Bacterial strains that did not display any lesion were considered as non-pathogenic. To confirm if the symptoms were caused by *P. ananatis*, bacteria were isolated from the region adjoining the symptomatic and healthy tissue on PA-20 semi-selective medium and incubated for 5–7 days at 28°C (Goszczynska et al., 2006). Presumptive colonies were further confirmed using a *P. ananatis*-specific assay as mentioned above (Goszczynska et al., 2006). Further, strain identity from randomly isolated colonies from *A. porrum* and *A. fistulosum × A. cepa* were confirmed by their DNA fingerprints using repetitive extragenic palindrome (rep)-PCR as previously described (Dutta et al., 2014a, b).

### Genome sequencing: Data filtering, draft genome assembly, and annotation

Genomic DNA was extracted utilizing the E.Z.N.A bacterial DNA kit Omega Bio-Tek (Norcross, GA). A 50 μl of DNA (50 ng/μl) per sample was used for library preparation as per the manufacturer’s instructions at Novogene Bioinformatics Technology Co. Ltd. (Beijing, China). Genomic DNA of each sample was randomly sheared into short fragments of about 200–400 base pairs (bp). The obtained fragments were subjected to library construction using the NEBNext^®^ DNA Library Prep Kit. After end repairing, dA-tailing, and further ligation with NEBNext adapter, the required fragments (in 200–400 bp size) were PCR enriched by P5 and indexed P7 oligos. The library was subsequently sequenced on Illumina NovaSeq 6,000 platform (Illumina Inc., San Diego, CA, United States). Pair-end sequencing were performed with the read length of PE 150 bp at each end. The raw fastq reads obtained were quality filtered. FastQC was used to assess the raw fastq files. Reads were filtered utilizing Trimmomatic (v. 0.36). The read data were filtered to remove low quality reads/bases and trimmed for reads containing primer/adaptor sequences using Trimmomatic’s ILLUMINACLIP paired end mode with seed mismatches set to two, palindrome clip threshold of 30, and a simple clip threshold of ten (Bolger et al., 2014). Further, all 5′ and 3′ stretches of ambiguous ‘N’ nucleotides were clipped to ensure high quality reads *via* setting both leading and trailing options to 3. The window size for sliding window was set to 4 with a required threshold of 30.

Trimmed data were re-assessed using FastQC and further used for genome assembly followed by pan-genome analyses. Further, all contigs ≤ 500 bp were removed using Seqtk (1.3). The cleaned reads were assembled using SPAdes (v. 3.15.3; Bankevich et al., 2012). Both the paired and unpaired data were used in assembly at default settings. The scaffolds of the respective 92 *P. ananatis* strains were annotated using Prokka (v. 1.14.5; Seemann, 2014). The resulting .gff files were used in the downstream pan-genome analysis.

### Average nucleotide identity, gene ontology assignment, and phylogenetic trees

Average nucleotide identity was determined using FastANI (Jain et al., 2018). KEGG gene ontology (GO) assignment was conducted using BioBam BLAST2GO pipeline (Götz et al., 2008; BioBam, 2022). A phylogenetic tree of single nucleotide polymorphisms (SNP’s) of core genome was generated utilizing PanSeq at default settings and RAxML alignment with 10,000 bootstrap replicates as per the previously reported methodologies (Laing et al., 2010; Stamatakis, 2014; Agarwal et al., 2021). The RAxML Boostrap random number used was 9,595, with parsimony random seed of 5,959. For Coinfinder and Roary plots, a tree was generated using FastTree 2.1.11 at default settings utilizing Roary’s core gene alignment (Price et al., 2009).

### Pan-genome, genome-wide associate studies, and gene coincidence of *Pantoea ananatis* (*N* = 92 strains)

All annotated genomes passed quality control and were used as inputs in ROARY (v. 3.12.0) at default settings that aided in generating a pan-genome with core and accessory genes. The complete pan-genome matrix in the form of presence and absence variant was used as inputs in SCOARY (v. 1.6.16). The GWAS analysis was conducted using the SCOARY program, which determined association between pangenomes and observed phenotypes (Page et al., 2015; Brynildsrud et al., 2016). This program was operated twice separately on each host plant of interest, once at default parameters, and a second time with a forced maximum value of *p* 0.05 across all testing parameters. For the gene association/dissociation analysis, complete pangenome of 92 *P. ananatis* strains were used as inputs for Coinfinder (v. 1.0.1). This program generated both gene-pair associations and dissociations with modification to association significance increased to a value of *p* of 0.1, and default settings (*p* = 0.05) for dissociation as previously described (Whelan et al., 2019). Direct comparisons of genetic sequences were performed using the Clustal Omega online server at default settings (Madeira et al., 2022).

### Tobacco infiltration assay for *Pantoea ananatis* strain (PNA 15-3) with putative Type III secretion system.

A single colony of *P. ananatis* strain PNA 15–3 (with putative T3SS) and a strain of *Pantoea stewartii* subsp. *indologenes* (20GA0713; positive control for T3SS) was suspended and grown overnight in modified Coplin medium (Asselin et al., 2018). Approximately 100 μl of the overnight culture was syringe-infiltrated into the tobacco leaf and the resulting infiltrated area was marked with a black marker. A sterile Coplin lab medium was used as a negative control. The symptom was observed at 48-h post inoculation (hpi) when the image was taken. This experiment was repeated twice.

## Results

### Phenotypic assessment of *Pantoea ananatis*: Red onion scale necrosis, foliar pathogenicity, and aggressiveness assay of on *Allium porrum* and *Allium fistulosum × Allium cepa*

Phenotyping of 92 *P. ananatis* strains displayed variability in the level of aggressiveness on both *Allium* spp. (Table 1). Variations in *P. ananatis* pathogenicity and aggressiveness on two *Allium* spp. were considerable (Figures 1A,B; Table 1). Strains screened in this study belonged to different isolation sources (Figure 1C; Table 1). Using the RSN disease phenotyping assay, we observed 61.3% (57/92) of *P. ananatis* strains displayed typical necrosis of red onion scale whereas 38.7%% (36/92) of strains did not cause necrosis (Figure 1D).

When *P. ananatis* strains were screened on *A. fistulosum × A. cepa*, 20.4% (19/92) and 44.1% (41/92) were found to be non-pathogenic and mildly aggressive, whereas 25.8% (24/92) and 9.7% (9/92) of the strains were identified as moderately aggressive and highly aggressive, respectively (Figure 1E). In contrast, on *A. porrum*, 45.7% (42/92) and 37% (34/92) of the strains were non-pathogenic and mildly aggressive, respectively. Interestingly, a much lower proportion of the strains; 14.1% (13/92) and 3.3% (3/92) identified as moderately aggressive and highly aggressive, respectively on *A. porrum* (Figure 1F). The percentage of strains that were pathogenic on *A. porrum* but non-pathogenic on *A. fistulosum × A. cepa* was only 2.1% (2/92). In contrast, 27% (25/92) of the strains that were pathogenic on *A. fistulosum × A. cepa* were non-pathogenic on *A. porrum*. Interestingly, 4.3% (4/92) of strains were highly aggressive on *A. porrum* but less aggressive on *A. fistulosum × A. cepa* whereas 9.7% (9/92) of strains were highly aggressive on *A. fistulosum × A. cepa* but less aggressive on *A. porrum*. Percentage of strains that were moderately to highly aggressive on both *Allium* spp. was 4.3% (4/92) whereas 18.8% (17/92) of the strains were non-pathogenic on both hosts tested. All the strains isolated from symptomatic *A. porrum* or *A. fistulosum × A. cepa* were identified as *P. ananatis* by recovery on PA-20 semi-selective medium and a *P. ananatis*-specific PCR assay as described above. *P. ananatis* colonies were not recovered from any of the negative control seedlings on PA-20 medium indicating no potential cross-contamination among the inoculated strains.

When comparing pathogenicity results with RSN results the 57 RSN-positive strains, 63.2% (36/57) of strains were pathogenic on both *A. porrum* and *A. fistulosum × A. cepa* whereas 0% (0/57) and 26.3% (15/57) of strains were only pathogenic on *A. porrum* or *A. fistulosum × A. cepa*, respectively. The remaining RSN-positive strains were non-pathogenic in the leaf tip necrosis assay on both hosts 10.5% (6/57). Among the RSN-negative strains, 41.6% (15/36) strains were non-pathogenic on both hosts, whereas 25% (9/36) were pathogenic on both hosts. Also, 5.5% (2/36) and 27.7% (10/36) of strains were pathogenic on only *A. porrum* and *A. fistulosum × A. cepa*, respectively. These results indicate that there is a discrepancy between the RSN phenotype and the foliar necrosis phenotype.

### The *Pantoea ananatis* pan-genome, architecture, and annotation

**Figure 2:**
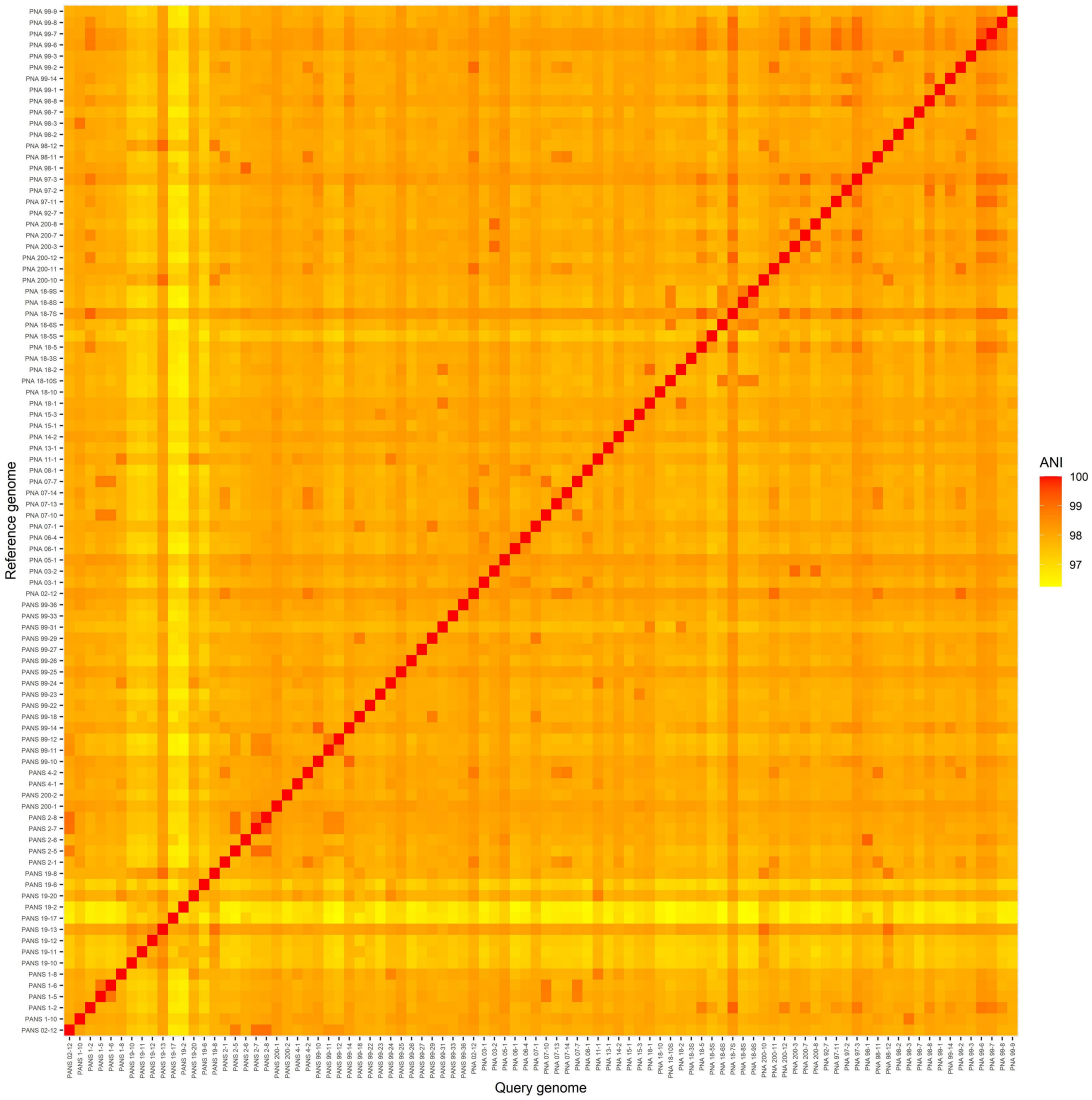
A heatmap of average nucleotide identity (ANI) comparisons of 92 *Pantoea ananatis* genomes. The smallest ANI comparison was between PNA 18-6S and PANS 19-17 with an ANI score of 96.25. The highest ANI comparison non-self-strain comparison was between PNA 99-6 and PNA 99-7 with a score of 99.41.

Post-sequencing, 1,594,092,228 raw reads were obtained and after stringent quality-filtering and trimming nearly 87% of the total reads (1,413,144,772 quality reads) were retained. The FastQC results indicated the sequence quality “passed,” as the majority of per-nucleotide and sequence qualities achieved high scores with no issues reported. For example: a good score can be ascertained with an average quality score of 30–40, with an exponentially increasing quality score distribution. Sequences that failed to incorporate into the final pangenome, or showed signs of contamination, were removed from the study. All *P. ananatis* sequences used in this study were submitted to NCBI (bio project PRJNA825576). Their corresponding accession numbers are listed in the Supplementary Table 1. The ANI matrix indicates that the strains investigated and utilized for genome analysis were indeed *P. ananatis* as the scores were more than 95% (Figure 2). The smallest ANI value was between PNA 18-6S vs. PANS 19-17 with an ANI score of 96.2% while the highest value was observed with PNA 99-6 vs. PNA 99-7 with a score of 99.4% (Figure 2).

**Figure 3:**
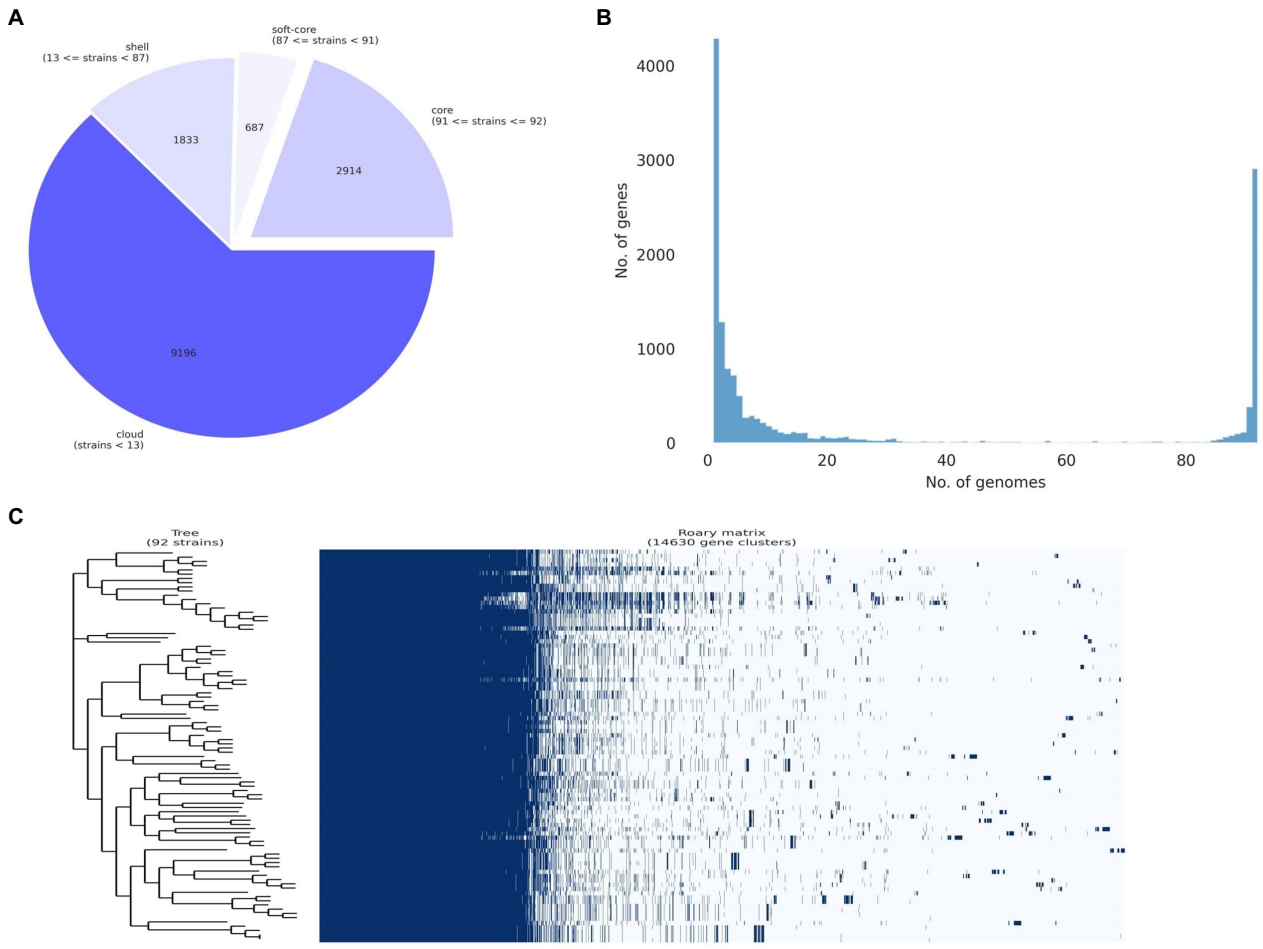
Pan-genome analysis of 92 *Pantoea ananatis* genomes. **(A)** Pie chart representation of pan-genome composition of *P. ananatis*. The core genome consists of 2,914 genes, the soft core 687 genes, the shell 1833 genes, and the cloud 9,196 genes for a total of 14,630 genes; **(B)** distribution of gene (cluster) sizes as a function of the number of genomes they contain displaying the partition of pan-genomic matrix into shell, cloud, soft-core and core compartments using ROARY outputs; and **(C)** pan-genome gene presence and absence matrix for 92 *P. ananatis* genomes and associated phylogeny of core gene alignment.

An overview of the final pangenome shows a core genome (occurs in 99% or more genomes, *N* > = 91) of 2,914 genes, a soft-core (occurs in 95–99% of genomes, *N* = 87 to 91) of 687 genes, a shell genome of 1833 genes (occurs in 15 to 95% of the genomes, *N* = 14–87), and a cloud genome (occurs in 0 to 15% of genomes, *N* = 0–14) of 9,196 genes for a total of 14,630 genes (Figure 3A). Details of the number of core and accessory genes contributed by each strain are shown in Figure 3B. A visual representation of the total presence and absence of genes within the pangenome where genomic differences in accessory components as well as the homogeneity of the core genome across the strains can be observed (Figure 3C). The phylogenetic tree produced that was used as input for both the gene-pair coincidence (GPC) analysis and the ROARY plots script is shown in Supplementary Figure 1.

The assignment of GO terms resulted in 19,323 annotations (Supplementary Figure 2). Among the genes annotated and assigned to biological processes (BP) within the pangenome, 3,828 are dedicated to cellular processes, 3,109 to metabolic processes, 930 to localization, 761 to biological regulation, 726 to the regulation of biological processes, 514 to the response to stimulus, 191 for signaling, 152 for the interspecies interaction between organisms, 101 for locomotion, 65 to viral processes, 46 for the negative regulation of biological processes, 43 for detoxification, 42 to developmental processes, 35 for positive regulation of biological processes, 36 for reproduction, 12 for nitrogen utilization, 5 for carbon utilization, 4 for multicellular organismal process, and one for immune system process (Supplementary Figure 2).

Among the genes that are assigned to molecular functions (MF), 3,182 are for catalytic activity, 2,713 for binding activity, 676 for transporter activity, 263 ATP-dependent activity, 252 with transcription regulator activity, 89 with molecular transductor activity, 82 with structural molecule activity, 37 with small molecule sensor activity, 34 for antioxidant activity, 30 with toxin activity, 23 with translocation regulation activity, 15 with molecular function activity, 12 for cytoskeletal motor activity, 6 for molecular carrier activity, and finally one assigned with nutrient reservoir activity. Among the genes assigned to cellular components, 2,894 are assigned as a cellular anatomical entity, 210 are protein-containing complexes, and 2 are virion components (Supplementary Figure 2). Further differentiation of these groups are available in the Supplementary Figure 3.

**Figure 4:**
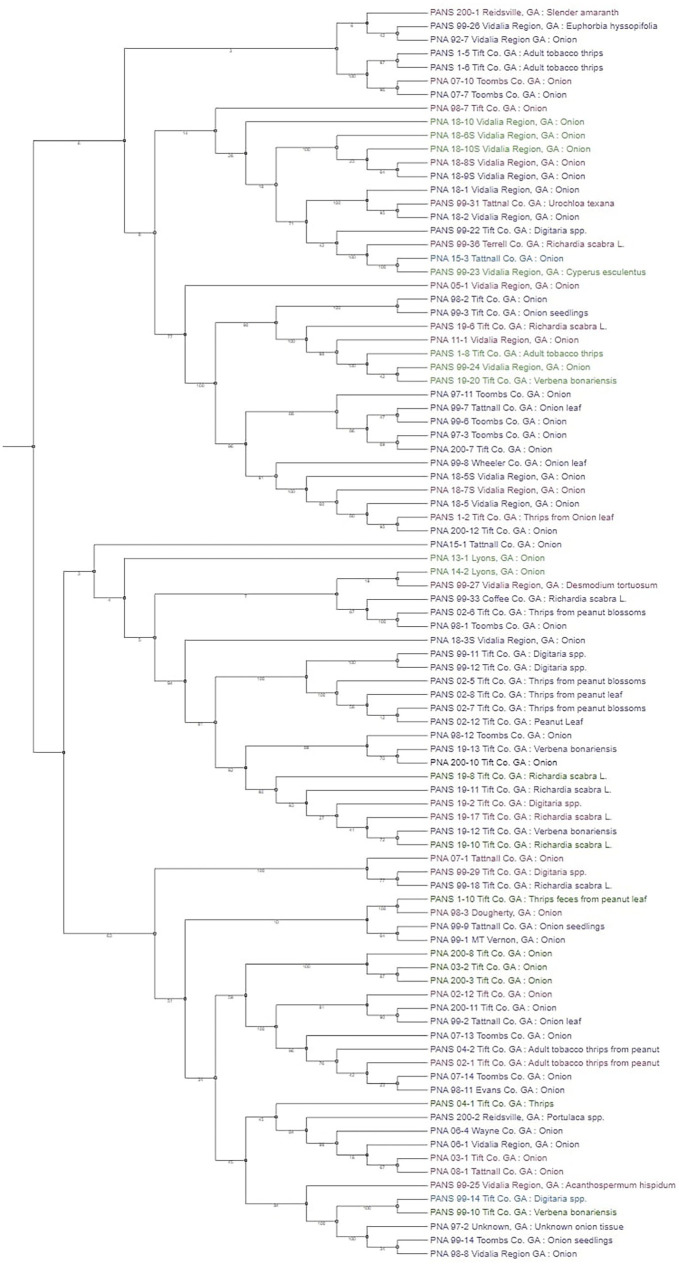
Phylogenetic tree based on core single nucleotide polymorphism (SNP) variants of genes among the *Pantoea ananatis* strains. Strains color coded in green are non-pathogenic on *Allium porrum* (cv. King Richard) and *A. fistulosum* × *A. cepa* (cv. Guardsman); strains that are color-coded in purple are pathogenic on both hosts; blue coded strains are pathogenic on *A. porrum* only; and pink coded strains are pathogenic on *A. fistulosum* × *A. cepa* only. Bootstrap values are shown on each branch after 10,000 iterations.

To determine the relationship between phylogeny of bacterial strains and their pathogenicity on *Allium* hosts, a phenotypic tree based on SNPs of core genes was constructed using RAxML and PanSeq (Figure 4) and is visually represented using the Interactive Tree of Life online tool (Letunic and Bork, 2016). When assessing the phylogenetic tree in its totality, it is difficult to determine a precise pattern except for strains from the same year of isolation tend to group together. This potentially indicates that these strains in the same group are genetically closely related. Despite this lack of obvious pattern in the overview of the phylogenetic tree, there are several clades where the terminal taxa are sorted based on their pathogenicity. Overall, these results provide support that changes in pathogenicity are not the result of strain lineage, but rather an expansive accessory genome.

### Genome wide association studies identify potential pathogenicity and virulence factors associated with *Pantoea ananatis* affecting *Allium porrum* and *Allium fistulosum × Allium cepa*

A pangenome utilizing ROARY was built, and the strength of gene association to the pathogenic phenotype on seedlings (*A. porrum* and *A*. *fistulosum × A. cepa*) was calculated using SCOARY. A total of 836 genes were found associated with RSN phenotype in *A. fistulosum × A. cepa* (*p* < =0.05).

Further, Benjamini-Hochberg correction (BHC) and Bonferroni correction were utilized in an attempt to avoid false positive associations (*p* < =0.05) and as a result only 50 genes were found significantly associated with the *A. fistulosum × A. cepa* foliar pathogenic phenotype. However, due to the GWAS results for *A. porrum* (discussed below) we found that the statistical association is not reflective of the biological role for some of the laboratory-based verified genes of interest. As such we included potential false positive associations in our analysis, but only focused on those that are shared between multiple analyses or those that seem to be similar to known gene clusters of interest. The GWAS results are listed in Supplementary File 1 in order of their statistical association. Within this total set of 836 genes, we found two divergent copies of phosphoenolpyruvate mutase (*pepM*) genes, annotated as “phosphonopyruvate hydrolase.” The first gene *hvrA/pepM* belongs to the previously described ‘HiVir’ cluster. Among the top twenty significantly associated genes with corrected *p*-values in *P. ananatis* affecting *A. cepa × A. fistulosum*, the HiVir cluster genes were found to be prominent (Supplementary file 1).

The second *pepM* gene belonged to a separate gene cluster (pgb) previously described by Polidore et al. (2021), which ranked at 451 based on naïve significance value. Five phosphatase genes, one gene related to chemotaxis, three related to virulence-region associated *virB*, and several genes in the previously described *alt* gene cluster were also associated with the pathogenicity (Jain et al., 2018; Stice et al., 2018, 2020). Two copies of the *fliC* gene, which encodes the flagellin monomer, were also found to be related with *P. ananatis’ pathogenicity* on *A. fistulosum × A. cepa* (Macnab, 2003; He et al., 2012). Flagellar motility has been previously observed to be important for onion leaf virulence (Weller-Stuart et al., 2017). Among the associated genes, we also screened for genes (annotated or hypothetical) to assess if they occur in clusters. Within the top 50 significantly associated genes we found at least five hypothetical gene clusters (group_4714–4719, group_3715–3726, group_5180–5182, group_5653–5660, group_5704–5778) as well as the HiVir gene cluster (Supplementary File 1).

Using *A. porrum* pathogenic strains, a total of 243 genes were found associated (naïve significance of *p* < = 0.05) with the pathogenic phenotype. However, none of the predicted genes were associated with the phenotype when the Bonferroni correction, and BHC were applied. When the naïve p-values were selectively screened for previously described genes known for pathogenicity and virulence in onion (HiVir genes, *alt*), we observed the HiVir cluster to be significantly associated with the phenotype; however, it ranked lower (rank: 91–101) compared to other annotated or hypothetical genes. The loss of statistically significant genes post-correction is likely due to the overall “potential resistance phenotype” this particular cultivar of *A. porrum* seems to display in our dataset. In following sections, we describe the biological relevance of the HiVir cluster for foliar pathogenicity in *A. porrum* and as such we believe that the naïve *p*-values are sufficient for further investigation despite the potential for false positives.

We also found significantly associated genes (*n* = 123), which were shared between the two hosts, with 48 of the top 50 associated genes (statistically significant post-correction) in *A. fistulosum × A. cepa* occurred in both GWAS results (Supplementary File 1). Some of the known genes that were shared between the *A. fistulosum × A. cepa* and *A. porrum* include the entire HiVir gene cluster, *pemK_2* (mRNA interferase), *soj* (sporulation initiation inhibitor protein), *parM* (Plasmid segregation protein), *umuD* (protein UmuD), *tibC* (glycosyltransferase), *ycaD* (uncharacterized MFS transporter), *dadA* (D-amino acid dehydrogenase 1), *frbC* (2-phosphonomethylmalate synthase), *amiD* (N-acetylmuramoyl-L-alanine amidase), and *rfbB* (dTDP-glucose 4,6-dehydratase). The genes that constitute the thiosulfinate tolerance cluster (*alt*) only appeared in *A. fistulosum × A. cepa* association with the phenotype with the following annotations; *xerC* (tyrosine recombinase XerC), *altA*/*nemA* (N-ethylmaleimide reductase), *gor* (glutathione reductase), *altJ/osmC* (peroxiredoxin OsmC), and *altD/trxA* (thioredoxin; Supplementary File 1). Genes that are members of the larger OVRA region, but not *alt*-specific genes in were also associated with the pathogenicity phenotype, and they include *rbsC* (ribose import permease protein RbsC), *rbsB* (ribose import binding protein), *rbsA* (ribose import ATP-binding), and *altD/trxA* (thioredoxin; Supplementary File 1). These results indicate that despite the lack of significance post-correction for the *A. porrum* GWAS results, there are still clusters of biological relevance for other *Allium* spp. might be important. Due to the proven role of the HiVir cluster in foliar pathogenicity, the remainder of this manuscript will focus on genes that are shared among multiple analyses, independent of the corrected significance values.

### Use of gene-pair coincidence for phenotype independent determination of pathogenicity and virulence factors

**Figure 5:**
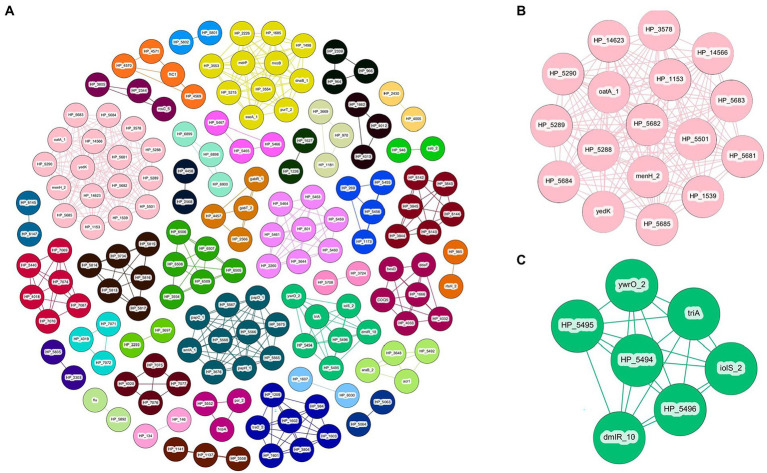
Coinfinder derived association of gene pairs from *Pantoea ananatis* genomes (*N* = 92) displaying various networks **(A)**. Gene-pair association networks for components 31 **(B)** and 11 **(C)** are extracted due to their association with foliar pathogenicity *for A. fistulosum* × *A. cepa.* Gephi was used to apply the Fruchtermann Reingold layout to the network (https://gephi.org/). For all node labels, “group_” was replaced by “HP_” (hypothetical protein) for legibility.

Gene-pair association of the *P. ananatis* pan-genome resulted in a total of 165 genes separated into 39 individual groups (Table 2; Supplementary File 2). Of the 165 genes, 45 genes are shared with the genes that are predicted based on GWAS for pathogenic phenotype on *A. fistulosum × A. cepa* and only two genes are shared with the genes that are predicted *via* GWAS for *A. porrum* pathogenicity (Table 2; Supplementary File 2). An overview of the associative Coinfinder output can be seen in Figure 5. Of the groups that also occurred on the GWAS analysis, 9/10 are saturated with genes that only associate with the pathogenic phenotype for *A. fistulosum × A. cepa*, and only one group is saturated with genes that associate with the pathogenic phenotype on *A. porrum*. These results indicate that in our pangenome there is a stronger associative pressure on genes that are specific to one host or the other, and there is no evidence for gene association between genes that associate for both hosts.

**Table 2:** List of gene-pair association components that contain genes shared with the predicted genes from the genome wise association studies (GWAS) results.

Gene-pair association and dissociation analysis				
Associated gene-pairs				
Component	GWAS correlation	Gene	Molecular function	Biological function
1	*A. fistulosum × A. cepa*	*group_3805*	–	–
		*rcsC_5*	ATP binding	Two-component regulatory system
		*group_2344*	–	–
3	*A. fistulosum × A. cepa*	*group_964*	–	–
		*group_966*	–	–
		*group_2339*	–	–
5	*A. fistulosum × A. cepa*	*rfaH_2*	DNA binding	Transcription/transcription antitermination
		*group_985*	–	–
11	*A. fistulosum × A. cepa*	*group_5494*	–	–
		*group_5495*	–	–
		*dmlR_10*	DNA-binding transcription factor	Regulation of transcription
		*ywrO_2*	NADPH dehydrogenase	Positive regulation of ion transport
		*group_5496*	–	–
		*iolS_2*	Oxidoreductase	Aldo-keto reduction
		*triA*	Hydrolase	Deamination
15	*A. fistulosum × A. cepa*	*group_1662*	–	–
		*group_4013*	–	–
		*group_4012*	–	–
16	*A. fistulosum × A. cepa*	acr1	Oxidoreductase	Lipid metabolic process
		araB_2	Ribulokinase	L-arabinose catalytic process
		group_5492	–	–
		group_3648	–	–
22	*A. fistulosum × A. cepa*	*group_3724*	–	–
		*group_5708*	–	–
24	*A. porrum*	*group_2568*	–	–
		*group_4458*	–	–
31	*A. fistulosum × A. cepa*	*oatA_1*	Acyltransferase	Lipopolysaccharide biosynthesis
		*group_3578*	–	–
		*group_1153*	–	–
		*group_14566*	–	–
		*group_5501*	–	–
		*group_5290*	–	–
		*yedK*	Peptidase/single-strand DNA binding	SOS response
		*group_5681*	–	–
		*group_5289*	–	–
		*group_14623*	–	–
		*group_5685*	–	–
		*group_5684*	–	–
		*group_5288*	–	–
		*menH_2*	Hydrolase activity	Menaquinone biosynthetic process
		*group_5682*	–	–
		*group_5683*	–	–
		*group_1539*	–	–
32	*A. fistulosum × A. cepa*	*flu*	Binding	Cell adhesion
		*group_5892*	–	–

**Figure 6:**
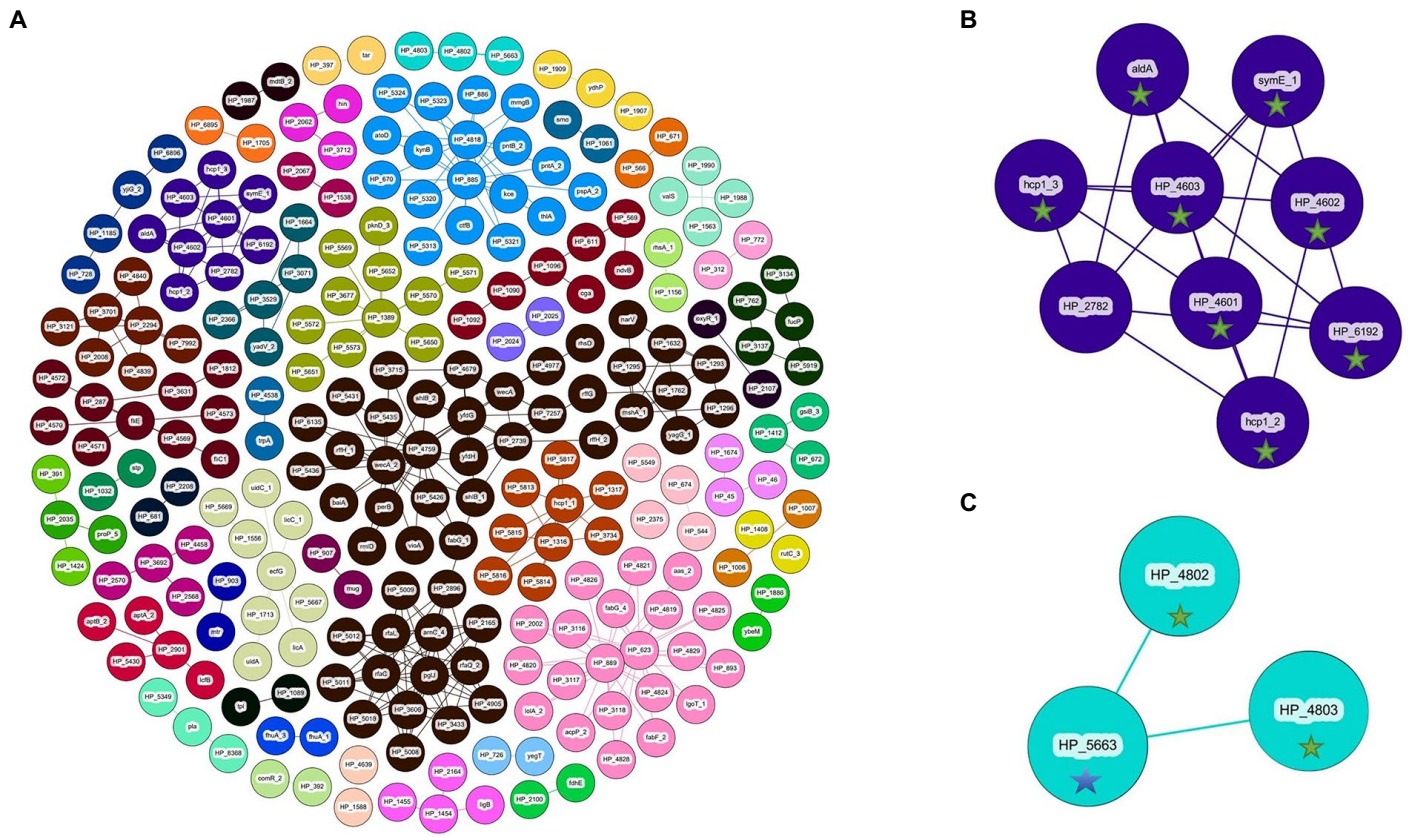
Coinfinder derived dissociation of gene pairs from *Pantoea ananatis* genomes (*N* = 92) displaying various networks **(A)**. Gene-pair dissociation networks for components 23 **(B)** and 37 **(C)** are extracted due to their with foliar pathogenicity *for A. fistulosum* × *A. cepa* (green star) or association with foliar pathogenicity for *A. porrum* (blue star). Gephi was used to apply the Fruchtermann Reingold layout to the network (https://gephi.org/).For all node labels, “group_” was replaced by “HP_” (hypothetical protein) for legibility.

Gene-pair dissociation of the *P. ananatis* pangenome resulted in 255 genes separated into 50 groups of dissociated genes (Table 3; Supplementary File 2). Of the 255 genes, 22 are shared with the genes associated with the pathogenic phenotype on *A. fistulosum × A. c*epa as predicted by GWAS, whereas only three genes are shared with the pathogenic phenotype on *A. porrum*. Components with dissociating gene-pairs that also occur on the GWAS output are summarized in Table 3. A full summary of the genes, their coincidence values, and their groups can be found in Supplementary File 2. An overview of the dissociative Coinfinder output can be seen in Figure 6. Among these groups, 4/6 show a dissociative relationship between genes that associate with the *A. fistulosum × A. cepa* disease phenotype and genes that do not associate with the pathogenic phenotype on either host. Finally, there is one group where there is a dissociative relationship between genes associated with the pathogenic phenotype in *A. fistulosum × A. cepa* or *A. porrum*, but not both (Table 3). These results indicate that there might be selective pressure leading to the evolution/acquisition of host-specific virulence factors rather than those that may be generally useful for virulence like the HiVir cluster.

**Table 3:** List of gene-pair disassociation components that contain genes shared with the predicted genes from the genome wise association studies (GWAS) results.

Gene-pair association and dissociation analysis				
Dissociated gene-pairs				
Component	GWAS correlation	Gene/group	Molecular function	Biological function
10	None	group_4840, group_3121, group_3701, group_2294	–	–
	*A. cepa × A. fistulosum*	group_7992, group_2008, group_4839		
17	None	group_611, group_1090, group_1096, group_1092	–	–
		cga	Carbohydrate binding	Carbohydrate metabolism
	*A. cepa × A. fistulosum*	group_569	–	–
		ndvB	Carbohydrate binding	Carbohydrate metabolism
21	*A. porrum*	group_2568, group_4458	–	–
	None	group_3692, group_2570		
23	None	group_2782	–	–
	*A. cepa × A. fistulosum*	group_4603, group_4602, group_4601, group_6192		
		hcp1_2	Family type IV secretion system effector	Toxin
		symE_1	DNA binding/RNA binding	RNA degradation
		hcp1_3	Family type IV secretion system effector	Toxin
		aldA	Aldehyde dehydrogenase	Varied catabolic processes
35	None	group_1295, group_1632, group_1293, group_1296, group_1762, group_7257, group_4977, group_5436, group_5426, group_2896, group_5435, group_5431, group_6135, group_2165, group_5008, group_3433, group_5012, group_5010, group_5009, group_5011, group_3715, group_4679, group_3606	–	–
		yagG_1	Symporter activity	Carbohydrate/sodium transport
		mshA_1	D-Inositol-3-Phosphate Glycosyltransferase activity	Mycothiol biosynthetic process
		rffG	dTDP-glucose 4,6-dehydratase Activity	Varied Biosynthetic Processes
		rffH_2	Glucose-1-phosphate Thymidylyltransferase activity	Extracellular polysaccharide biosynthetic process
		narV	Nitrate reductase activity	Aerobic respiration/nitrate assimilation
		rmlD	dTDP-4-dehydrorhamnose reductase activity	dTDP-rhamnose biosynthetic process/polysaccharide biosynthesis/O-Antigen biosynthesis
		perB	DNA binding	Regulator
		baiA	NAD+ binding	Protein homotetramerization
		vioA	dTDP-4-amino-4,6-dideoxy-D-glucose transaminase activity	Lipopolysaccharide biosynthetic process
		fabG_1	3-Oxoacyl-[acyl-carrier-protein] reductase (NADH) Activity	Fatty acid elongation
		rffH_1	Glucose-1-phosphate Thymidylyltransferase Activity	Extracellular polysaccharide biosynthetic process
		arnC_4	Undecaprenyl-phosphate 4-deoxy-4-formamido-L-arabinose Transferase Activity	Polysaccharide biosynthetic process
		rfaQ_2	Glycosyltransferase activity	–
		rfaC	Lipopolysaccharide heptosyltransferase activity	Lipopolysaccharide core region biosynthetic process
		rfaL	Ligase activity	Lipopolysaccharide core region biosynthetic process
		pglJ	Hexosyltransferase activity	Protein N-linked glycosylation via asparagine
		yfdH	Glycosyltransferase activity	–
		shlB_2	–	Protein transport
		rfaQ_2	Glycosyltransferase activity	–
		rfaC	Lipopolysaccharide heptosyltransferase activity	Lipopolysaccharide core region biosynthetic process
		rfaL	Ligase activity	Lipopolysaccharide core region biosynthetic process
		pglJ	Hexosyltransferase activity	Protein N-linked Glycosylation via Asparagine
		yfdH	Glycosyltransferase	–
		wecA	Glycosyltransferase activity	O Antigen biosynthetic process
	*A. fistulosum ×* A. cepa	group_4905, group_2739, group_4759	–	
		shlB_1	Putative exported adhesin activator	Protein transport
		rhsD	–	Cellular response to sulfur starvation
		wecA_2	Glycosyltransferase activity	O Antigen biosynthetic process
37	*A. porrum*	group_5663	–	–
	*A. fistulosum ×* A. cepa	group_4803, group_4802		

### Comparative genomics of strains with *Allium* species-specific pathogenicity

Five strains were chosen based on their species-specific pathogenicity on *Allium* hosts. The strains PNA 15-3, PANS 99-14 were pathogenic on *A. porrum*, but non-pathogenic on *A. fistulosum × A. cepa* and were all RSN-negative. The strains PNA 07-10, PNA 07-1, and PNA 05-1 were all pathogenic on *A. fistulosum × A. cepa* and RSN-positive, but non-pathogenic on *A. porrum* (Table 1). Gene presence and absence were compared manually among these strains. All strains possessed the HiVir cluster except for the strain PNA 15-3. The absence of this cluster is the likely cause for the strain’s inability to cause foliar lesions on *A. fistulosum × A. cepa*, and necrosis on onion scale.

The strain PNA 15-3; however, carries genes that indicate the presence of a type III secretion system, a virulence pathway that uncommon in *P. ananatis* (Supplementary Table 2). When comparing this type-III secretion system to those found in Kirzinger et al. (2015), it appears to show similarities with PSI-1b. Attempts to align this sequence to the type III secretion system found in *P. stewartii* subsp*. indologenes* indicated low sequence similarity. When PNA 15-3 was inoculated into tobacco leaf panels, no hypersensitive response was observed (Supplementary Figure 4). We found 43 genes proximal to each other surrounding the *stcC* gene in PNA 15-3. A total of 35 genes in the cluster were annotated as hypothetical proteins. The other eight genes were annotated as: *oleC* (olefin beta-lactone synthetase), *gacA* (response regulator GacA), *mxiA* (protein MxiA), *hrcN* (type III secretion ATP synthase HrcN), *spaP* (surface presentation of antigens protein SpaP), *spaQ* (surface presentation of antigens protein SpaQ), *yscU* (yop proteins translocation protein U), *sctC* (type 3 secretion system secretin), and *dctD* (C4-dicarboxylate transport transcriptional regulatory protein DctD). Further, we utilized NCBI database nucleotide BLAST to query the type III secretion system sequence at default values for the *P. anantis* taxid in the WGS database. We observed an 88% similarity with over 98% query coverage for PANS 99-23, PANS 99-26, PANS 200-1, PNA 86-1, UMFG54 (JACAFO010000015.1), NRRL B-14773 (JACEUA010000002.1), and DE0584 (VDNR01000019.1). Using NCBI database nucleotide blast at default values for the *P. anantis* taxid in the nr nucleotide collection, we observed an 88% identity with LCFJ-001 (CP066803.1) and FDAARGOS_680 (CP054912.1) chromosomal sequences.

The strains PNA 07-10, PNA 07-1, and PNA 05-1 shared several genes that do not occur in PNA 15-3, or PANS 99-14. These genes include the *alt* cluster, a cluster of 12 genes with three annotations (*argT_3*: lysine/arginine/ornithine-binding periplasmic protein, group_2282: ureidoglycolate lyase, and *dapL*: LL-diaminopimelate aminotransferase), a cluster of 12 genes (*virB_2*: virulence regulon transcriptional activator VirB, *uspA_2*: universal stress protein A, *galE_2*: UDP-glucose 4-epimerase, *ybjJ_2*: inner membrane protein YbjJ, *nudK_3*: GDP-mannose pyrophosphatase NudK, group_5271: phosphorylated carbohydrates phosphatase, *mtnP*: S-methyl-5′-thioadenosine phosphorylase, *gph_2*: phosphoglycolate phosphatase, *arnB_2*: UDP-4-amino-4-deoxy-L-arabinose—oxoglutarate aminotransferase, *arnB_3*: UDP-4-amino-4-deoxy-L-arabinose—oxoglutarate aminotransferase, *perA*: GDP-perosamine synthase, and *iolG_5*: inositol 2-dehydrogenase/D-chiro-inositol 3-dehydrogenase). These strains share 10 more genes in common, with 7 annotations (*bepF:* efflux pump periplasmic linker BepF, group_5443: adaptive-response sensory-kinase SasA, *phoP_3*: alkaline phosphatase synthesis transcriptional regulatory protein PhoP, *parA_2*: plasmid partition protein A, *yedK_1*: SOS response-associated protein YedK, *ppaC*: putative manganese-dependent inorganic pyrophosphatase, and *crcB_2*: putative fluoride ion transporter CrcB). Of the 60 genes shared between these strains, none of them are associated with the *A. porrum* disease phenotype and 58 genes are associated with the *A. fistulosum × A. cepa* disease phenotype. The two genes that were not associated with the *A. fistulosum × A. cepa* disease phenotype are annotated as hypothetical genes. Of these 60 genes, only one appears in the gene pair dissociation component 30 as the hypothetical gene “tar” dissociating with group_397 (Supplementary Table 2).

Apparent gene clusters shared by the *A. porrum* pathogenic PNA 15-3 and PANS 99-14 include a moderate gene cluster of 7 hypothetical genes and *caf1M* (chaperone protein Caf1M), another gene cluster of 7 hypothetical genes as well as 4 annotated genes (*amiD_3*: N-acetylmuramoyl-L-alanine amidase AmiD, *yraI_2*:putative fimbrial chaperone YraI, *htrE_2*: outer membrane usher protein HtrE, *fimC*: chaperone protein FimC). Half of a third cluster is shared between the two strains, with one hypothetical, with one hypothetical protein and three annotated proteins (group_5495: HTH-type transcriptional regulator PgrR, *iolS_2*: aldo-keto reductase IolS, *ywrO_2*: general stress protein 14). The final cluster consists of 10 genes without annotations, and group_7087 (replicative DNA helicase). Of the 39 shared genes, none appeared to be associated with the foliar pathogenic phenotype on *A. porrum* whereas 28 genes did appear to be associated with the foliar pathogenic phenotype for *A. fistulosum × A. cepa.* Furthermore, of these genes, none appeared in any gene-pair dissociation output. However, 19 of these genes appeared in the gene-pair association output within components 9 (*N* = 1), 11 (*N* = 5), 31 (*N* = 3), 37 (*N* = 2), 38 (*N* = 3), and 39 (*N* = 4; Supplementary Table 2).

**Figure 7:**
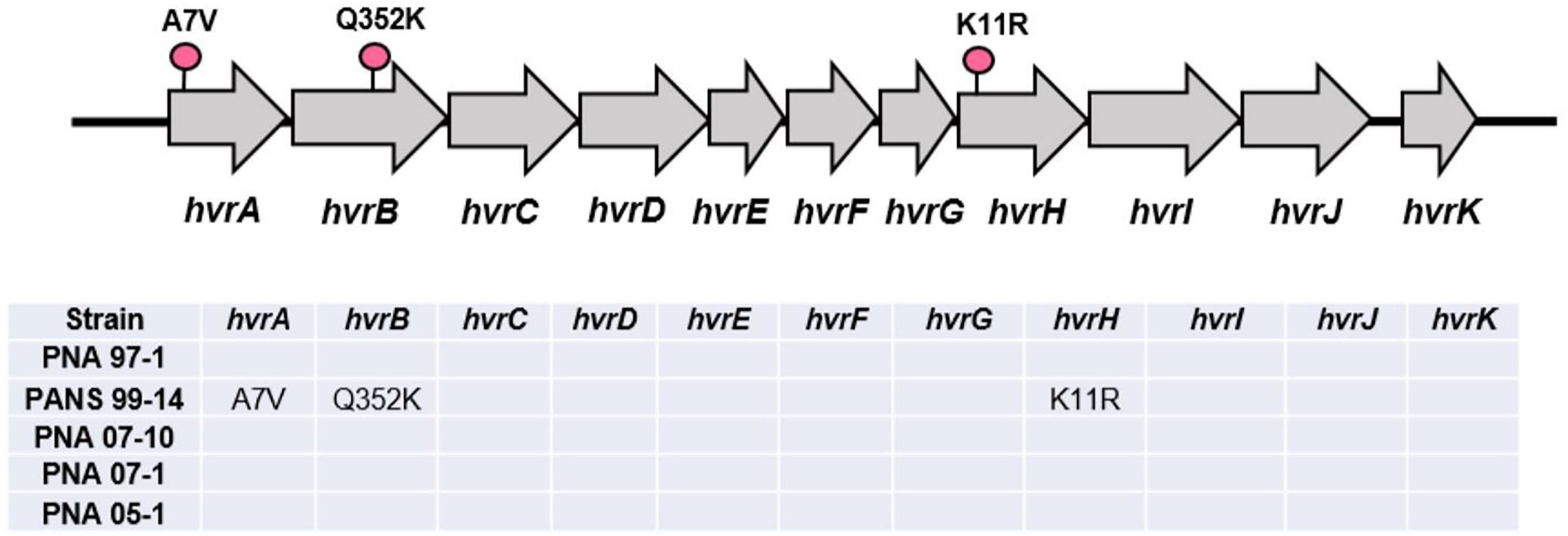
Graphical representation of the HiVir cluster with single nucleotide polymorphisms (SNPs) determined *via* direct comparison with the PNA 97-1 “wildtype.” Unique missense SNPs are coded with a pink pin. A short table below shows SNP comparisons between the *A. fistulosum* × *A. cepa* pathogenic strains (PNA 07-10, PNA 07-1, and PNA 05-1) against the *A. porrum* pathogenic strains (PNA 15-3 and PANS 99-14).

The HiVir gene cluster found in the RSN-positive, *A fistulosum × A. cepa* pathogenic strains PNA 07-10, PNA 07-1, and PNA 05-1 did not contain any single nucleotide polymorphism (SNP) compared to that of the RSN-positive, wild type *P. ananatis* PNA 97-1 (Figure 7). However, three unique SNPs that resulted in missense mutations were identified in the RSN-negative, *A. porrum* pathogenic strain PANS 99-14. These mutations included alanine (A) to valine (V) change in amino acid position 7 in *hvrA* (*pepM*) gene, glutamine (Q) to lysine (K) change in amino acid position 352 in *hvrB* gene and, lysine (K) to arginine (R) change in amino acid position 11 in *hvrH* gene. These variant SNPs could be associated with disruption of the pantaphos pathway and loss of necrosis-associated phenotypes (Figure 7).

### Description of a “pgb” gene cluster in *Pantoea ananatis*

Comparative genome analysis focusing on only strains that were pathogenic on *A. porrum*, but non-pathogenic on *A. fistulosum × A. cepa* (PNA 15-3, PANS 99-14) vs. strains (PANS 99-11, PANS 99-12, PNA 06-4, PNA 99-9) that were pathogenic and highly aggressive on both hosts identified another *pepM* gene, which appeared to be a member of a secondary phosphonate biosynthetic cluster (Table 4). In 8/92 of *P. ananatis* strains (PANS 02-12, PANS 99-31, PANS 200-2, PANS 2-5, PANS 2-7, PANS 2-8, PANS 99-11, and PANS 99-12) there was a putative phosphonate biosynthetic cluster with 14 genes and a total length of approximately 19,000 bp. This cluster showed high sequence similarity to the pgb-cluster mentioned in Polidore et al. (2021), which was not responsible for generating onion-bulb rot symptoms. In our annotations, the left-flank of the cluster begins with a prophage integrase *intS* and is followed by the *pepM* phosphoenolpyruvate mutase (5′–3′ 900 bp long). The following *cpdA* is a 3′,5′-cyclic adenosine monophosphate phosphodiesterase *cpdA* (3′–5′ 771 bp). The third component of the cluster is *fabG* encoding 3-oxoacyl-[acyl-carrier-protein] reductase FabG3 (765 bp 3′–5′). The fourth gene is a phosphonopyruvate decarboxylase, *aepY* (1,176 bp, 3′–5′). The fifth gene of the cluster is *phnW* encoding for 2-aminoethylphosphonate-pyruvate transaminase (1,095, 5′–3′). The sixth component is *asnB1* encoding putative asparagine synthetase [glutamine-hydrolyzing] (1,758 bp, 3′–5′). Following *asnB2* is spsI1, encoding for Bifunctional IPC transferase and DIPP synthase (771 bp 5′–3′). The gene *asd1* follows *spsl1* that encodes aspartate-semialdehyde dehydrogenase (1,056 bp 5′–3′). The ninth component of the cluster is the MFS 1 transporter (1,227 bp, 5′–3′). The tenth component is *glyA1*, a serine hydroxymethyltransferase (1,359 bp, 5′–3′). The CDP-alcohol phosphatidyltransferase is the eleventh gene (600 bp 5′–3′). The twelfth gene is *spsI2*, a glucose-1-phosphate adenylyl/thymidylyltransferase Bifunctional IPC transferase and DIPP synthase (720 bp, 5′–3′). The thirteenth gene is *aspC1*, aspartate aminotransferase (1,173 bp, 5′–3′). The fourteenth gene is UDP-2,3-diacylglucosamine diphosphatase (762 bp, 3′–5′). Following the UDP-2,3-diacylglucosamine diphosphatase is another transposase. An interesting observation of this phosphonate cluster is the inclusion of phosphonopyruvate decarboxylase directly within the set of genes. This characteristic is unique when comparing it to the HiVir. The phosphonopyruvate decarboxylase has been described to play a critical role in the generation of phosphonates *via* the stabilization of the PEP mutase reaction (Supplementary Table 3; Kirzinger et al., 2015).

**Table 4:** Composition and annotation of the HiVir and the pgb gene clusters in *Pantoea ananatis*.

HiVir gene cluster	Annotations	pgb gene cluster	Annotations2
hvrA	**Phosphoenolpyruvate phosphomutase**	pepM	**Phosphoenolpyruvate phosphomutase**
hvrB	FMN-dependent oxidoreductase (nitrilotriacetate monooxygenase family)	cpdA	3',5'-cyclic adenosine monophosphate phosphodiesterase CpdA
	Monooxygenase	fabG	3-oxoacyl-[acyl-carrier-protein] reductase FabG
hvrC	Homocitrate synthase NifV	aepY	phosphonopyruvate decarboxylase
	2-Isopropylmalate synthase	phnW	2-aminoethylphosphonate--pyruvate transaminase
hvrD	3-Isopropylmalate/(R)-2-methylmalate dehydrataselarge subunit	asnB	Putative asparagine synthetase [glutamine-hydrolyzing]
hvrE	3-Isopropylmalate dehydratase small subunit	spsI1	Bifunctional IPC transferase and DIPP synthase
hvrF	Methyltransferase	asd	Aspartate-semialdehyde dehydrogenase
hvrG	Acetyltransferase (GNAT) family protein		**MFS 1 transporter YcxA**
hvrH	ATP-grasp domain containing protein	glyA	Serine hydroxymethyltransferase
hvrI	**MFS transporter**		CDP-alcohol phosphatidyltransferase
	Macrolide efflux protein A	spsI2	glucose-1-phosphate adenylyl/thymidylyltransferase
hvrJ	Hypothetical protein		Bifunctional IPC transferase and DIPP synthase
hvrK	Flavin reductase	aspC	Aspartate aminotransferase
			UDP-2,3-diacylglucosamine diphosphatase

### Presence and absence of *Alt*, HiVir, pgb, gene clusters

**Figure 8:**
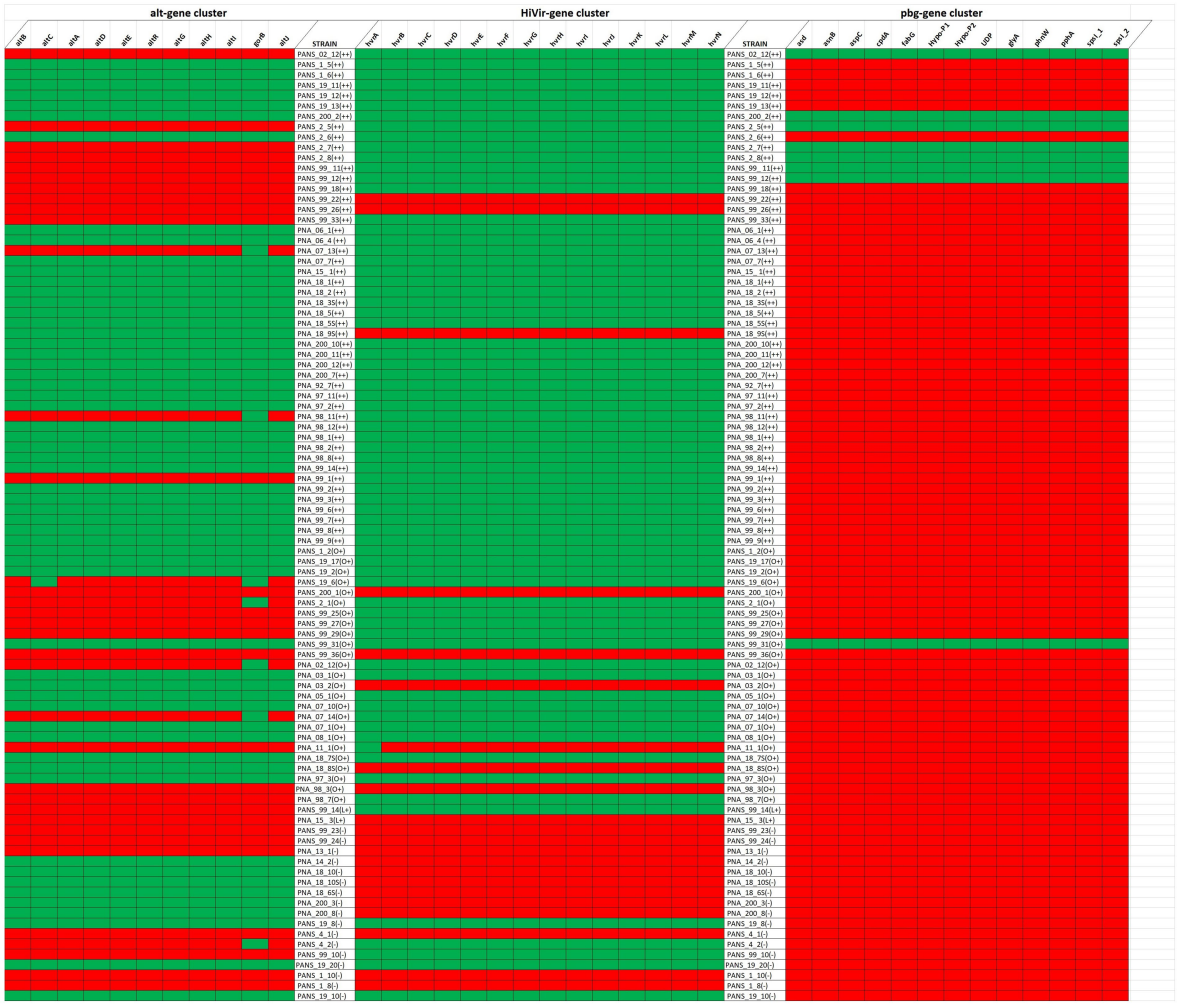
Presence and absence of the Alt (left), HiVir (middle), and the pgb cluster (right) genes within the genome of *Pantoea ananatis* based on their foliar pathogenicity on *Allium porrum* (cv. King Richard) and *A. fistulosum* × *A. cepa* (cv. Guardsman). Green and red represent presence and absence of gene, respectively for each gene cluster evaluated. The foliar pathogenicity phenotype and associated aggressiveness is represented as “++” if the strain is pathogenic on both hosts, “L+” if pathogenic only on *A. porrum*, “O+” if pathogenic only on *A. cepa* × *A. fistulosum*, and “–” if pathogenic on neither host.

Of the 92 tested strains, 35 do not contain a complete *alt* gene cluster while the remaining 57 do possess the entire gene cluster. Of the 92 tested strains, 22 lacked a complete HiVir cluster and the remaining 70 strains possessed the entire gene cluster. Of the strains that produced foliar lesions, 45 had both *alt* and HiVir clusters, whereas 20 strains had only the HiVir gene cluster. Of the 92 tested strains, 3 strains only had *alt* whereas 7 strains lacked both gene clusters. Some foliar lesions were formed by the 7 strains (PANS 99-22, PANS 99-26, PANS 200-1, PANS 99-36, PNA 98-3, PNA 11-1, and PNA 15-3) that lacked both clusters, however the lesions varied in size and consistency between replicates. The strains that lacked both gene clusters were also RSN-negative. Of the 7 strains, showed some degree of foliar lesions on *A. cepa × A. fistulosum*; however, PNA 15-3 showed moderately aggressive lesion length on *A. porrum* but did not produce any foliar lesion on *A. cepa × A. fistulosum*. Of the 92 tested strains, only 8 strains contained the pgb cluster, while 84 strains lacked it (Figure 8).

### Comparison of phosphonate biosynthetic clusters, pgb vs. HiVir in *Pantoea ananatis*

When comparing the pgb cluster against the HiVir cluster, only the annotated *pepM* and MFS transporter are found to be common features (Table 4). In both clusters, the phosphoenolpyruvate mutase occurs first, with the MFS transporter being at the center of the cluster (5′–3′: 9th gene in pgb and 5′–3′: 9th in HiVir). There are no other shared annotated genes between the clusters. However, sequence alignment using Clustal Omega revealed 48.3% similarity between *pepM* from HiVir and pgb cluster. Similarly, the MFS transporters from HiVir and pgb clusters displayed 47.6% sequence similarity.

### Role of *pepM* gene in the pgb biosynthetic cluster

**Figure 9:**
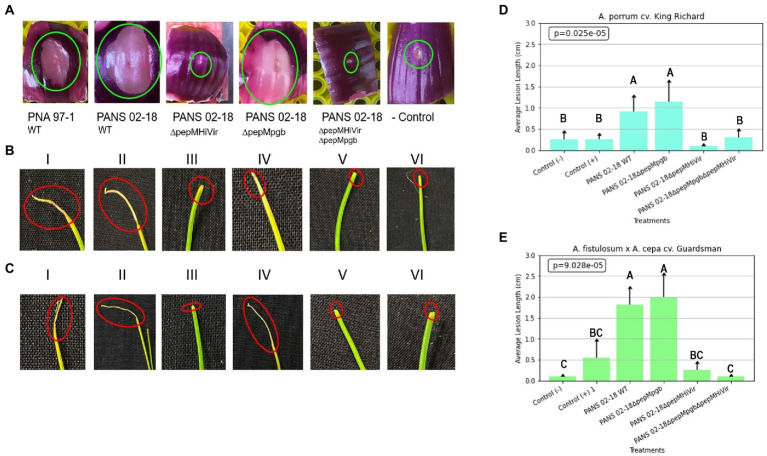
Role of HiVir and pgb biosynthetic clusters in foliar pathogenicity of *Pantoea ananatis* on *Allium* species. Panel **(A)**: Red scale necrosis phenotype observed with the wild-type and mutant strains of *P. ananatis*; panel **(B)**: Foliar pathogenicity assay on leek (*Allium porrum* cv. King Richard) seedlings with the wild-type and mutant strains of *P. ananatis*; and **(C)** Foliar pathogenicity assay on Japanese bunching onion (*A. fistulosum* × *A. cepa* cv. Guardsman) seedlings with the wild-type and mutant strains of *P. ananatis*. Green and red circles indicate red scale necrosis and foliar necrosis, respectively. Panels **(D)** and **(E)** display the results of the mean lesion length with standard error in *A. porrum* and *A. fistulosum* × *A. cepa* upon inoculation with the wild-type and mutant strains of *P. ananatis*. The strains utilized in the experiments mentioned above include positive control strain PNA 97-1, positive control PANS 99-11 (a known aggressive strain on leek), PANS 02-18_ΔpepMHiVir_, PANS 02-18_ΔpepMpgb_, and PANS 02-18_ΔpepMΔpepMpgb_. Seedlings and red scale inoculated with sterile water comprised negative controls. Data presented here is the mean of two independent experiments. Letters on the bars indicate mean separation with LSD *p* < 0.05.

Based on the RSN assay the wild-type strain (PANS 02-18) and the single *pepM* mutant strain in the pgb cluster (Δ*pepM*_pgb_) produced considerably large necrotic areas on red-onion scale compared to the single *pepM* mutant strain in the HiVir cluster (Δ*pepM*_HiVir_; Figure 9A). Based on the seedling pathogenicity assay, the single *pepM* mutant strain in the HiVir cluster (Δ*pepM*_HiVir_) had significantly lower necrotic lesion length on both *Allium* hosts compared to the wild-type strain (PANS 02-18) and the single *pepM* mutant strain in the pgb cluster (Δ*pepM*_pgb_; Figures 9B,C). In both hosts, the deletion of *pepM* gene in the pgb cluster did not significantly affect the foliar lesion length compared to the wild-type strain (Figures 9D,E). While the double mutant strain where *pepM* genes were deleted in both the HiVir and the pgb clusters (Δ*pepM*_HiVir_Δ*pepM_pgb_*) appears to have displayed a higher average lesion length than that of the single mutant strain (Δ*pepM*_HiVir_) on *A. porrum* (Figures 9B,D). This result is surprising; however, we found no statistical significance between the mean lesion length of the Δ*pepM*_HiVir_Δ*pepM_pgb_* vs. the Δ*pepM*_HiVir_ strains (Figure 9D). In *A. fistulosum* × *A. cepa*, the lesion lengths did not differ significantly between the double mutant strain and the single mutant strain (Δ*pepM*_HiVir_).

## Discussion

### Pathogenicity and aggressiveness of *Pantoea ananatis* phenotyping on *Allium porrum* and *Allium fistulosum*

Phenotyping of 92 *P. ananatis* strains displayed variability in the level of aggressiveness in both *Allium* spp. A considerably higher percentage of strains were either non-pathogenic or less aggressive on *A. porrum* (82.6%) than on *A. fistulosum × A. cepa* (65.1%). Also, a considerable percentage of strains were moderately or highly aggressive on *A. fistulosum × A. cepa* (34.9%) compared to *A. porrum* (17.4%). Interestingly, when a subset of less-aggressive strains (on both *Allium* spp.) was previously assayed on onion seedlings they were moderately-to-highly aggressive on onion (Stice et al., 2018; Agarwal et al., 2021). These observations potentially indicate that both *A. porrum* and *A. fistulosum × A. cepa* are inherently less susceptible to *P. ananatis* compared with the typical bulb onion. Also, when *A. porrum* and *A. fistulosum × A. cepa* were compared to each other, the former tends to show less severe symptoms compared to the later. However, we acknowledge that only one cultivar of each *Allium* spp. was evaluated, and it is possible that other cultivars or varieties of these hosts might show a range of susceptibility to *P. ananatis*. One aspect of these observations might be in part explained by the genetic nature of the *A. fistulosum × A. cepa*. cv. Guardsman itself. Being a hybrid between bunching onions and the typical bulb onion, it may be reasonable to expect *A. fistulosum × A. cepa* to be more susceptible to *P. ananatis* strains that were collected from symptomatic *A. cepa* tissues. However, without deeper genetic investigation of this hybrid we cannot predict for certain if any susceptibility-related genes or phenotypes were inherited. The *P. ananatis* culture collection used in this study also favors areas where onions are grown such as the Vidalia region and Tift County in Georgia, United States, which may provide a bias for aggressive bacterial strains on cultivars that are hybridized with *A. cepa*. Despite this, we recovered some strains that were more aggressive on *A. porrum* than *A. fistulosum × A. cepa*. Examples of these are PANS 99-11, PANS 99-12, and PNA 06-4 (Table 1).

### *HiVir* gene cluster, previously identified as critical for red onion scale necrosis and *Allium cepa* pathogenicity, is also important for foliar pathogenicity in *Allium porrum*, *Allium fistulosum* × *Allium cepa*

Of the tested strains 56 showed a positive reaction to the RSN assay, while other strains (*n* = 36) did not. Most of the strains that were pathogenic on *A. fistulosum* × *A. cepa* were also able to cause necrosis on red onion scale. Based on the previous reports, HiVir is important for RSN-positive phenotype and foliar necrosis in onion, and it is likely that foliar lesions on *A. fistulosum* × *A. cepa* is also governed by the same gene cluster. Similarly, in *A. porrum*, a trend between RSN-positive phenotype and foliar pathogenicity was observed with majority of the strains. Further mutational analysis also indicated that HiVir is important for foliar pathogenicity on both *A. fistulosum* × *A. cepa* and *A. porrum*. However, we identified several strains that did not follow this trend. For example, while possessing a complete HiVir cluster and being RSN-positive, the PANS 19-8, and PANS 19-10 strains were unable to cause foliar lesions on *A. porrum* or *A. fistulosum × A. cepa*. This indicates that either mutation in their nucleotides/SNPs or other/alternative pathogenicity factors might be involved with this group of strains. Despite the importance of the HiVir cluster, there were 10 strains that seemed to cause foliar lesions despite lacking a complete HiVir cluster (PANS 99-22, PANS 99-26, PNA 18-9S, PANS 200-1, PANS 99-36, PNA 03-2, PNA 11-1, PNA 18-8S, PNA 98-3, and PNA 15-3). These observations indicate that other pathogenicity factors might be involved in these strains. Consistent with prior observations by Polidore et al. (2021), we also observed that the pgb cluster is not important for foliar pathogenicity in these *Allium* spp. The PANS 02-18 Δ*pepM*_pgb_ and wild-type strains displayed similar foliar pathogenicity phenotype in *A. porrum* or *A. fistulosum × A. cepa.*

### Pan-genome of *Pantoea ananatis* and GWAS for foliar pathogenicity phenotype on *Allium* spp

In this study, we generated a pan-genome of 92 *P. ananatis* strains where we identified a conserved core genome of 2,914 genes, with a larger accessory genome of 9,196 genes. Earlier pan-genome reports identified similar values of core genes, with varying numbers of *P. ananatis* strains used for the analysis (De Maayer et al., 2014; Sheibani-Tezerji et al., 2015; Stice et al., 2018; Agarwal et al., 2021). In this work, however, we used a larger set of accessory genes compared to previously observed pan-genome study by Agarwal et al. (2021). We observed 6,808 cloud genes compared to 9,196 cloud genes in our current study. This discrepancy is like due to the use of larger number of diverse strains in this study compared to Agarwal et al. (2021) and is a reasonable increase for an open pangenome (Costa et al., 2020). Due to the cosmopolitan nature of *P. ananatis* and the extensive host range, it is entirely plausible that the bacterium would have a sizable pangenome when comparing populations from diverse hosts (De Maayer et al., 2014). It may be prudent to utilize a larger collection of strains from non-*allium* hosts for further pan-genomic assessments, where accessory genes may aid in resolving these strains further down beyond species taxonomic classification. In further attempts to discern the low statistical strength of the gene-associations, we noticed that several genes with high homology were assigned separate annotation tags. It is possible that these tags could artificially inflate the pangenome, leading to a weakened statistical association (Supplementary Figure 4). Even if the pangenome was artificially inflated, the GWAS results indicate significant association of genes that are known virulence factors (such as the HiVir cluster), as well as putative virulence factors that were found previously to be associated with foliar pathogenicity in *A. cepa* (Agarwal et al., 2021).

We identified 244 genes that were significantly associated with foliar pathogenicity in *A. porrum* whereas 836 total genes were associated with foliar pathogenicity in *A. fistulosum × A. cepa,* with 50 genes displaying significant agreement between naïve, Bonferroni, and BHC corrections for foliar pathogenicity in *A. fistulosum × A. cepa*. Among the genes associated with virulence in two hosts, 123 genes were shared. For both hosts, the HiVir cluster was found within the top-100 significantly associated genes. The occurrence of this cluster grants some additional credibility to other genes that show stronger significance with phenotypic association. Further investigation is required to determine the validity of these genes that are truly associated with the pathogenicity phenotype, or are errors from random sampling of the genome. In addition to this, most of the genes that occurred in the GWAS output apart from the HiVir cluster are annotated as hypothetical and would require further characterization to determine their relevance. Among these 123 genes, 48 of the top 50 statistically significant genes associated with the pathogenic phenotype in *A. fistulosum × A. cepa* were also associated with the pathogenic phenotype in *A. porrum*. While this alone is not enough to state their relevancy, it lends some credibility that these hypothetical genes may be useful as general virulence factors for the *Alliums* spp. and should undergo downstream mutational analysis to assess their functions.

Some of the genes with non-hypothetical annotations that were shared between the *A. fistulosum × A. cepa* and *A. porrum* include the entire HiVir gene cluster (some listed as hypothetical), *pemK_2* (mRNA interferase), *soj* (sporulation initiation inhibitor protein), *parM* (Plasmid segregation protein), *umuD* (UmuD, translesion DNA polymerase subunit), *tibC* (glycosyltransferase), *ycaD* (uncharacterized MFS transporter), *dadA* (D-amino acid dehydrogenase 1), *frbC* (2-phosphonomethylmalate synthase), *amiD* (N-acetylmuramoyl-L-alanine amidase), and *rfbB* (dTDP-glucose 4,6-dehydratase). Upon manual investigation of the local *pemK_2* region there seems to be a repeating pattern of seven genes, three flanking to the left, and four on the right. Utilizing BLAST for these gene sequences against the total gene sequences available for our *P. ananatis* strains shows that the pemK_2 region appears frequently throughout the entirety of the pangenome (Supplementary Table 5). The *pemK* gene is a known factor in toxin/antitoxin systems that are vital for bacterial competition and function (Lee et al., 2012; Klimina et al., 2013; Poluektova et al., 2017). Unfortunately, there is little information within the literature pertaining to the potential diversity and utility of *P. ananatis* toxin/antitoxin systems, including *pemK*. Without functional analysis, it is not possible to determine whether it is an *Allium*-specific virulence factor as opposed to a coincidental gene cluster, or if the annotation provided is correct. We also found an antitoxin gene, *higB* that was associated with *A. fistulosum × A. cepa* pathogenicity. Despite the lack of information, the region may be a valuable target for a toxin/antitoxin system within our *P. ananatis* strains from Georgia. Another annotated gene of interest includes *tibA*, an adhesin/invasion autotransporter. The sporulation initiation inhibitor protein, soj, is noted as possess a “centromere-like function involved in forespore chromosome partitioning inhibition of Spo0A activation” in *Bacillus subtilis* (Kunst et al., 1997). *P. ananatis* is not a spore forming bacteria; however, the inclusion of this gene with other genes that are similarly annotated for DNA manipulation may indicate that there is a requirement for maintaining genetic stability. The genes that constitute the “thiosulfinate tolerance; *alt* cluster,” only appeared in the *A. fistulosum × A. cepa* GWAS output with their Uniprot annotations of *xerC* (tyrosine recombinase XerC), *altA*/*nemA* (N-ethylmaleimide reductase), *gor* (glutathione reductase), *altJ*/*osmC* (peroxiredoxin OsmC), and *altD*/*trxA* (thioredoxin). Non-*alt* members of the OVRA region include *rbsC* (ribose import permease protein RbsC), *rbsB* (ribose import binding protein), and *rbsA* (ribose import ATP-binding).

Overall, GWAS was able to determine genes associated with foliar necrosis in *A. fistulosum × A. cepa* and *A. porrum* hosts. Follow-up experiments will test the validity of these hypothetical and annotated gene clusters for their relevance in pathogenicity and virulence in *A. fistulosum × A. cepa* and *A. porrum* hosts.

### Comparative genomics of strains pathogenic on *Allium porrum* but non-pathogenic on *Allium fistulosum × Allium cepa* against strains that are pathogenic on *Allium fistulosum × Allium cepa* but non-pathogenic on *Allium porrum*

By comparing strains that were pathogenic on only *A. fistulosum × A. cepa* or *A. porrum* we hoped to significantly reduce the background noise (non-relevant genes from accessory) that may potentially result from the extensive *P. ananatis* pan-genome. Here we found several genes that belonged to strains that were only pathogenic to one host or the other, as well as a few interesting gene clusters.

One of the gene clusters of interest appears to be saturated with genes with annotated function of a typical type III secretion system. When using nucleotide blast in NCBI against the nr and whole genome sequence database, there were several other *P. ananatis* strains that shared a high consensus to the sequence (*N* = 11; in NCBI). Most of the strains in NCBI with an annotated type III secretion-system were isolated in Georgia, USA (*N* = 7; in NCBI) with two strains from onions and five strains from weeds. The remaining four strains with potential type III secretion-system were not isolated from in Georgia, USA. Further work is required to assess if the annotations are correct and investigate their role in onion pathogenicity.

Among the clusters shared by the *A. fistulosum × A. cepa* pathogenic strains PNA 07-10, PNA 07-1, and PNA 05-1, we found the *alt* cluster, two larger gene clusters (*N* = 12 genes, *N* = 22 genes), and two small gene clusters (*N* = 7 genes, *N* = 3 genes). Of the total 60 shared genes, none of them are on the *A. porrum* GWAS output, and 58 appeared in the *A. fistulosum × A. cepa* GWAS output. Only one gene appeared in the gene pair dissociation component 30 as the hypothetical gene tar that dissociated with group_397 (Supplementary File 4). Among the clusters shared by the *A. porrum* pathogenic strains; PNA 15-3 and PANS 99-14, we observed three gene clusters with 7, 8, and 11 genes each. Of these 39 genes, 28 were identified through GWAS as associated with the disease phenotype for *A. fistulosum × A. cepa*, but not associated with the disease phenotype in *A. porrum* (Supplementary Table 2). None of these genes appeared in the gene-pair dissociation output. However, 19 of these genes were found within components 9 (*N* = 1), 11 (*N* = 5), 31 (*N* = 3 genes), 37 (*N* = 2 genes), 38 (*N* = 3 genes), and 39 (*N* = 4 genes) in the gene-pair association analysis (Supplementary Table 2).

While the inclusion of the *alt* cluster in the *A. fistulosum × A. cepa* strains PNA 07-10, PNA 07-1, and PNA 05-1 is unsurprising, as they were isolated from symptomatic onions, its absence from PNA 15-3 is unexpected. The strain PNA 15-3 was isolated from symptomatic onion bulbs, and we would expect the presence of *alt* cluster as it aids in colonization of the onion bulb (Stice et al., 2020). The strain PANS 99-14 was isolated from an asymptomatic *Digitaria* spp. and may not need to rely on an *alt* cluster to survive in this environment. Despite this, both strains were able to generate a lesion on the *A. porrum* foliar tissue, and both strains failed to produce a positive result in the red-onion scale necrosis assay. These results indicate that some of the shared genes between these strains may aid in increased fitness in the *A. porrum* foliar environment that is not present in the *A. cepa* bulb tissue, or the *A. fiustulosum* × *A. cepa* foliar tissue. The large percentage of these shared genes (19/39) occur in the gene-pair association output, seems to suggest that these genes occur together at a higher frequency than others. Much like how the *alt* cluster provides protection to *P. ananatis* in the thiosulfinate-rich in bulb, it is possible that some of these clusters may provide protection to bacteria in the diverse *Allium* foliar environments.

We also aligned the HiVir gene clusters of *A. fistulosum × A. cepa* pathogenic (PNA 05-1, PNA 07-1 and PNA 07-10) and *A. porrum* pathogenic strains (PNA 15-3, and PANS 99-14) against each other and against the wild type *P. ananatis* strain PNA 97-1. No single nucleotide polymorphism (SNP) leading to missense mutation was identified in the RSN positive, *A. fistulosum × A. cepa* pathogenic strains (PNA 05-1, PNA 07-1 and PNA 07-10 strains). However, several missense mutations were present in the *hvr*A, *hvrB* and *hvrH* genes of the RSN negative, *A. porrum* pathogenic strain (PANS 99-14). TThese mutations were found only in *hvr* genes of PANS 99-14 but not in the *hvr* genes of the RSN positive strain (PNA 97-1). According to Metcalf and Van Der Donk (2009) and Polidore et al. (2021), *hvrA* and *hvrB* genes encode enzymes that are essential for the proposed phosphonate-toxin ‘pantaphos’ biosynthesis pathway. It is thus possible that the production of phosphonate toxin is compromised by these mutations. However, functional analysis needs to be conducted to confirm the impact of these mutations. In the case of RSN negative PNA 15-3, the strain lacked HiVir cluster but was still able to cause foliar lesions on *A. porrum*. It is possible that the pathogenicity of *A. porrum* in RSN negative PNA 15-3 and PANS 99-14 strains might possible be mediated by the genes other the genes in the HiVir cluster. Our results are limited by the number of strains used in the direct comparison; however, they do seem to indicate that there could possibly other Allium-specific virulence factors other than this phosphonate gene cluster (HiVir). These results are further visualized by aligning two figures (Figures 4, 8), where the pattern of the presence and absence of HiVir and *alt* gene clusters alone are not sufficient to map host pathogenicity (Supplementary Figure 5).

### Gene-pair coincidence

In this work, we utilized gene-pair coincidence as a supporting methodology to predict genes in *P. ananatis* are potentially relevant in *Allium* spp. pathogenicity. Hypothetically, genes that are important for survival in pathogenic bacteria should associate throughout a pangenome as their co-occurrence is beneficial for survival. Likewise, gene combinations that compromise survival in specific environments should dissociate with each other as natural selection selects against non-optimized populations. In this work, we hypothesized that the utilization of gene-pair coincidence should provide a phenotype-independent method of validation for the predicted genes from the pangenome *via* the phenotype-dependent GWAS methodology.

Our gene-pair association analysis generated 39 networks with a total of 165 individual genes, where two common genes were associated with the pathogenic phenotype in *A. porrum* and 45 common genes were associated with the pathogenic phenotype in *A. fistulosum × A. cepa*. In gene networks with genes that associated with the pathogenic phenotype (networks: 1, 3, 5, 11, 15, 16, 22, 24, 31, 32), the entire component is found in the GWAS result, indicating that these pathogenicity-associating genes are evolutionarily are potentially associated with each other via evolutionary process in our pangenome. Of these associated gene pair networks, none of the genes are associated with the disease phenotype for both hosts, only one or the other. Component 24 is unique in that only genes with the pathogenic phenotype in *A. porrum* were present. It is possible that these genes provide a unique advantage to overcome *A. porrum* host resistance. Unfortunately, both genes are annotated as hypothetical. The genes found in components 1, 3, 5, 11, 15, 16, 22, 24, 31, and 32 may be more useful for bacterial survival in *A. fistulosum × A. cepa* as opposed to being general virulence factors. Gene-pair association has provided support for further investigation of several potential gene groups that could potentially be harder to distinguish utilizing GWAS alone. Surprisingly, neither the HiVir gene cluster nor the *alt* cluster appear in the associative network. We would assume that both the gene clusters should co-occur together as they are relevant in bacterial pathogenicity and virulence in *A. cepa* and are quite prevalent in the pangenome. It could possibly due to limited number of genomes used in this study as compared to the number of strains/genomes needed to close the pan-genome. Another explanation could be the way the ROARY/Coinfinder organizes gene information and it is possible that the known virulence factors were omitted from pairwise analysis due to the lack of orthologous gene families. To determine if the issue was caused by noise because of genes not present in the gene clusters, we conducted analysis after removing individual genes from the ROARY csv file. However, this only strengthened resulting p-values, but did not improve the overall results. As such a larger sample size of strains with a comprehensive accessory genome could be included to better support GPC and GWAS results.

Our gene-pair dissociation analysis generated 50 gene-pair networks with a total of 255 genes, where only three genes were associated with the pathogenic phenotype in *A. porrum* and 22 genes associated with the pathogenic phenotype in *A. fistulosum × A. cepa*. Here, dissociation is dominated by networks where only a fraction of the dissociated genes also associated with the pathogenic phenotype in either of the Allium hosts. Dissociation networks 10, 17, 23, and 35 contained genes that associated with the pathogenic phenotype in *A. fistulosum × A. cepa* but dissociated with the genes that did not associate with the pathogenic phenotype in *A. fistulosum × A. cepa*. Dissociation network 37 is particularly interesting in that it showed dissociation between one hypothetical gene that associated with the pathogenic phenotype in *A. porrum*, and two genes that associated with the pathogenic phenotype in *A. fistulosum × A. cepa*. Whether these hypothetical genes have contrasting functions are yet to be evaluated; however, it is worth investigating if these genes play roles in host-pathogen-environment interactions. Dissociation network 21 is the only network where two genes that associated with the pathogenic phenotype in *A. fistulosum × A. cepa* dissociated with two genes that did not associate with the pathogenic phenotype in either hosts. These results are expected as genes that associate with the foliar pathogenic phenotype should dissociate with genes that do not associate with the same phenotype. Here we did not observe the HiVir or the alt genes dissociated with other genes, indicating that these gene clusters do not compete with each other in *P. ananatis*’ pangenome.

Again, these observations provide some level of confidence that the genes being predicted in the GWAS output are playing a role that enables them to be associated with the foliar pathogenicity phenotype on both *Allium* hosts. The diversity of GPC and their occurrence in the phenotype-dependent analysis enforce the assumption made previously that there could be several mechanisms of causing disease in *Allium* species other than phosphonate-based toxins.

## Conclusion

In this study, we have used two parallel analytical approaches of phenotype-dependent and phenotype-independent ‘informatics approaches to predict *Allium*-specific pathogenicity factors. These methodologies were able to identify genes of interest that may be associated in *Allium* pathogenicity in *P. ananatis.* We concluded that several genes are associated with foliar pathogenicity in both *A. porrum* and *A. fistulosum × A. cepa* as determined by our foliar necrosis assay. Among our strains, the presence of the HiVir cluster in most of the cases correlates with the pathogenic foliar phenotype in these *Allium* species. However, it may not be the only factor driving pathogenicity across these *Allium* species. The previously reported HiVir gene cluster was important for foliar pathogenicity in these *Allium* species as determined by its association with the disease phenotype as well as mutational analysis. When comparing strains that were pathogenic on only one host, *A. porrum* or *A. fistulosum × A. cepa*, we found several genes that are exclusive to one host or the other. The GWAS/GPC approach can be deployed in other host species where *P. ananatis* causes disease and may provide a more thorough understanding of what complex interactions are relevant for the phenotype of interest, assuming the sample size is sufficient. More “informatics” approaches will be needed to mine deeper insights into the identification of the hypothetical/novel genes, as well as the appropriate functional analysis for validation. It is likely that a combination of integrated approaches utilizing transcriptomics, proteomics, and functional analysis of genes and gene clusters may potentially provide information on the role of critical genes that *P. ananatis* utilize to infect diverse *Allium* hosts.

## Data availability statement

The original contributions presented in the study are publicly available. This data can be found at: NCBI, PRJNA825576.

## Author contributions

BM and BD conceived the project. BM performed the bioinformatics analyses and compiled the manuscript. BM, BD, and GS designed and finalized the manuscript. BD planned the project, secured extramural funds, and revised and submitted the manuscript. BD, BK, GA, SS, and RG: resources, supervision, and writing—review and editing. All authors contributed to the article and approved the submitted version.

## Funding

This study was supported in part by resources and technical expertise from the Georgia Advanced Computing Resource Center, a partnership between the University of Georgia Office of the Vice President for Research and Office of the Vice President for Information Technology. This work was partially supported by the Specialty Crop Block Grant AWD00009682.

## Conflict of interest

The authors declare that the research was conducted in the absence of any commercial or financial relationships that could be construed as a potential conflict of interest.

## Publisher’s note

All claims expressed in this article are solely those of the authors and do not necessarily represent those of their affiliated organizations, or those of the publisher, the editors and the reviewers. Any product that may be evaluated in this article, or claim that may be made by its manufacturer, is not guaranteed or endorsed by the publisher.
