# Supplementary figures and images for: Genome-wide association and dissociation studies in Pantoea ananatis reveal potential virulence factors affecting Allium porrum and Allium fistulosum × Allium cepa hybrid
Source: Front Microbiol. 2023 Feb 2;13:1094155. doi: 10.3389/fmicb.2022.1094155 (PMC9933511; doi:10.3389/fmicb.2022.1094155)

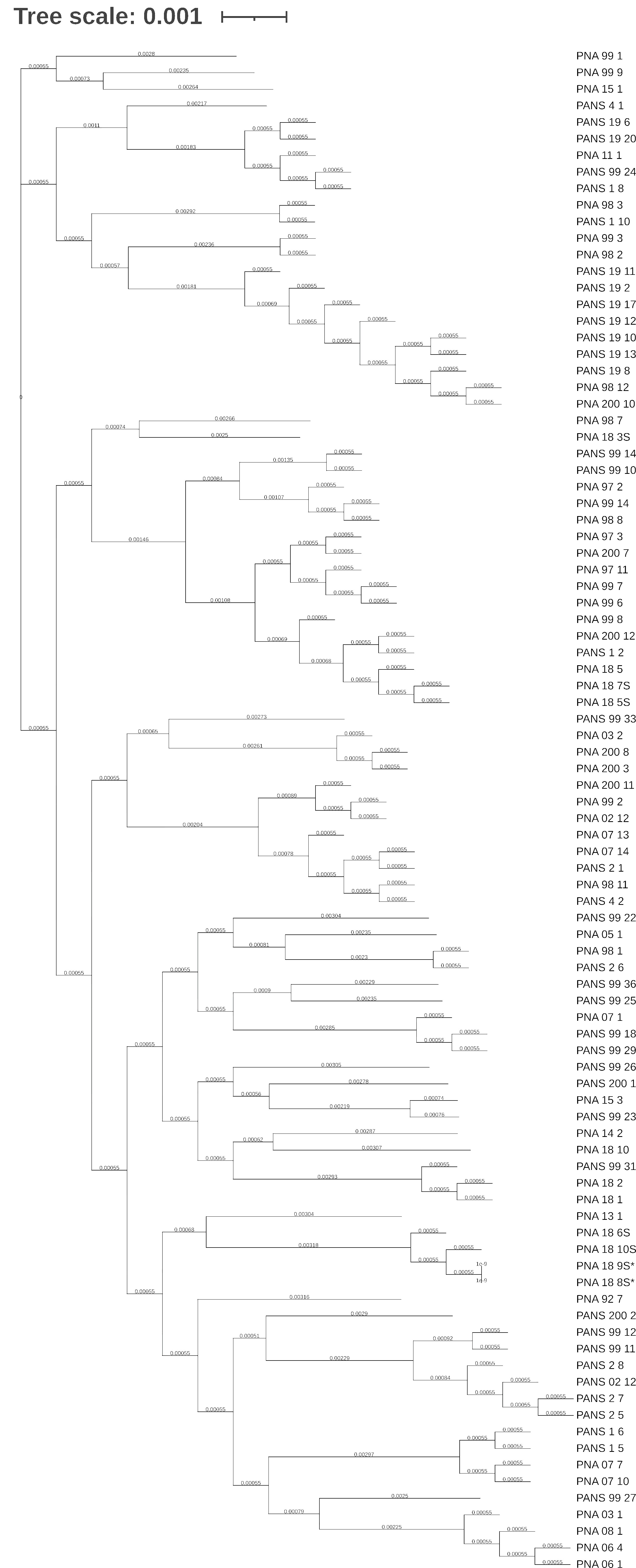

Supplement: Supplementary file 12 [file Image_1.JPEG]

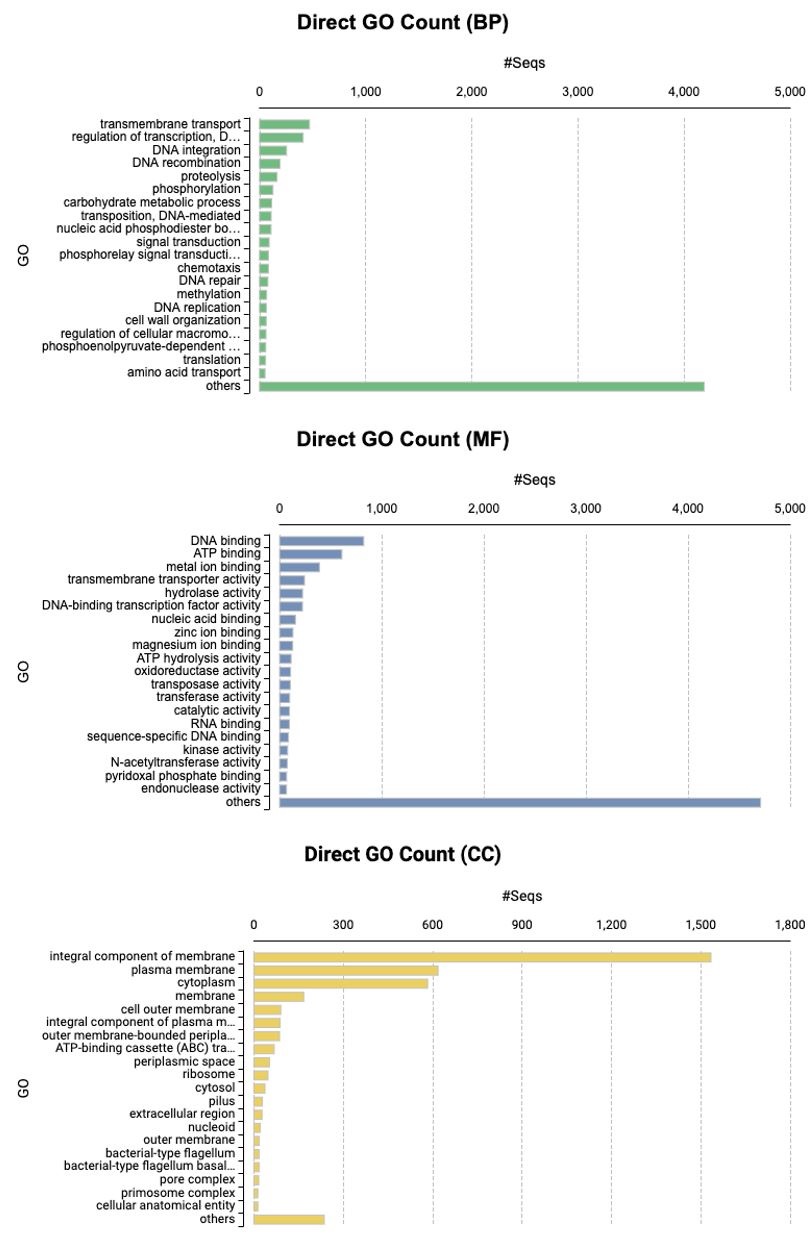

Supplement: Supplementary file 13 [file Image_2.JPEG]

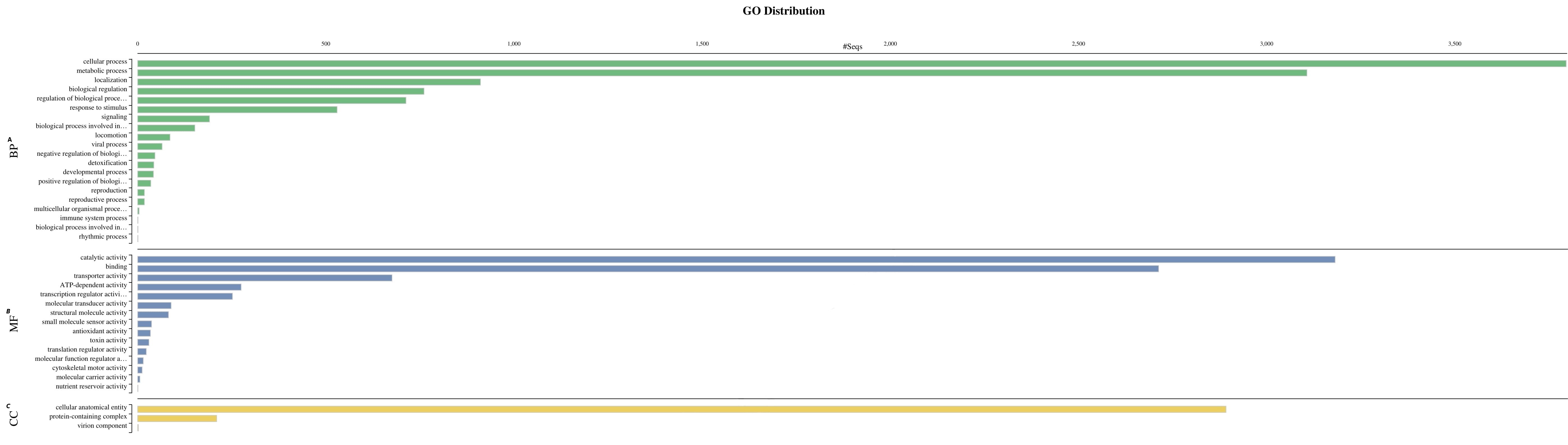

Supplement: Supplementary file 14 [file Image_3.JPEG]

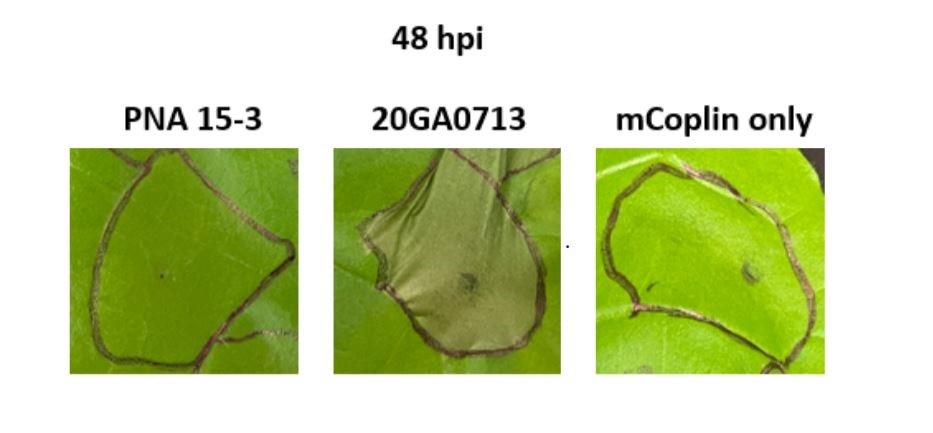

Supplement: Supplementary file 15 [file Image_4.JPEG]

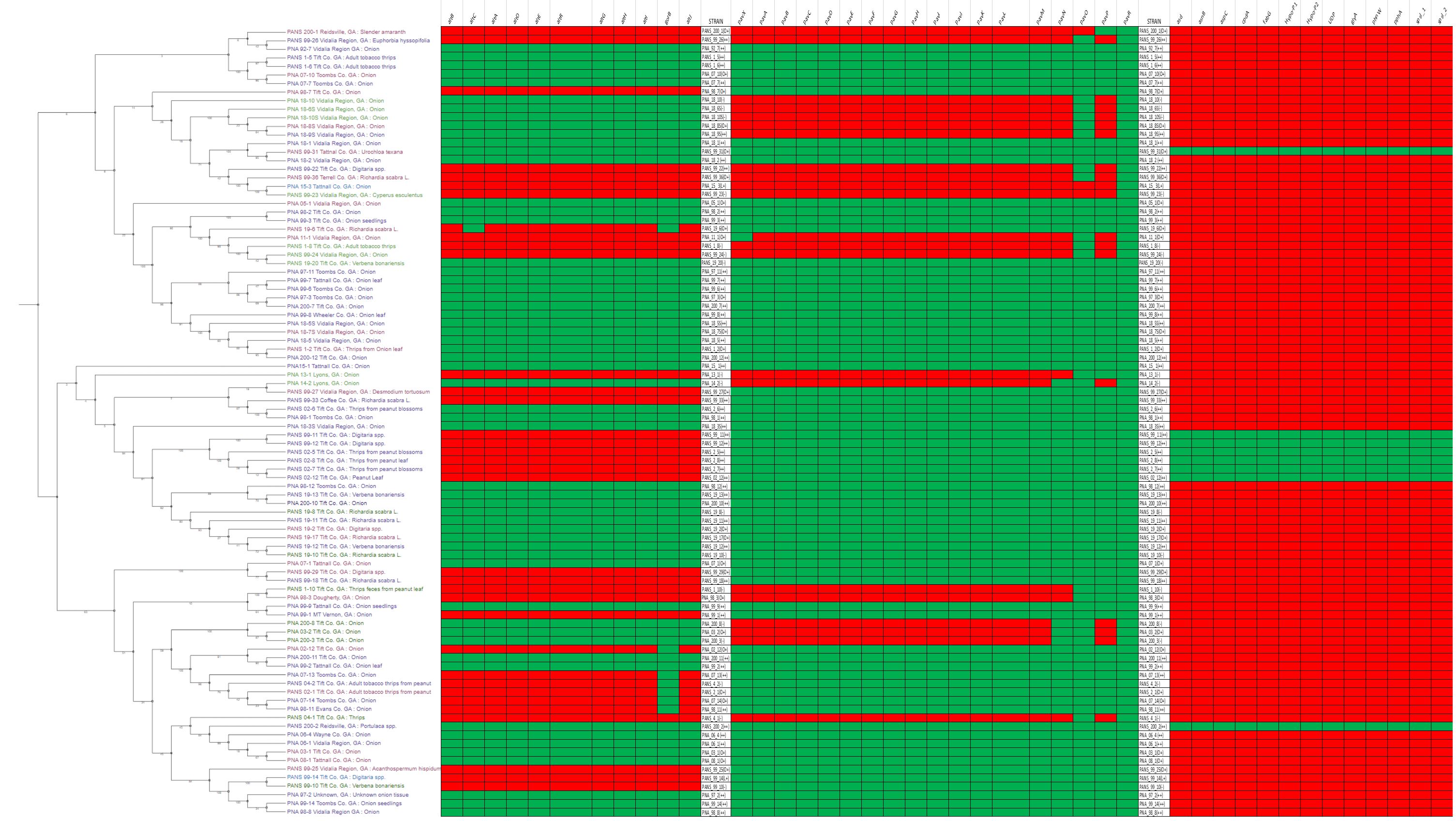

Supplement: Supplementary file 16 [file Image_5.JPEG]
